# The Value of Subjective Olfactometry as a Predictive Biomarker of Neurodegenerative Diseases: A Systematic Review

**DOI:** 10.3390/life14030298

**Published:** 2024-02-23

**Authors:** Laia Ramos-Casademont, Daniel Martin-Jimenez, Brenda Villarreal-Garza, Serafín Sánchez-Gomez, María Amparo Callejon-Leblic

**Affiliations:** 1 Department of Otolaryngology, Vic University Hospital, 08500 Vic, Spain; lramosc.girona.ics@gencat.cat; 2 Rhinology Unit, Department of Otolaryngology, Head and Neck Surgery, Virgen Macarena University Hospital, 41009 Seville, Spain; divan.martin.sspa@juntadeandalucia.es (D.M.-J.); bvilgar@alu.upo.es (B.V.-G.); serafin.sanchez.sspa@juntadeandalucia.es (S.S.-G.); 3 MED-EL, Centro Empresarial Euronova, 28760 Madrid, Spain; 4 Biomedical Engineering Group, University of Seville, 41004 Seville, Spain

**Keywords:** Alzheimer’s disease, neurodegenerative disease, olfaction, olfactory disorder, Parkinson’s disease, smell, subjective olfactometry

## Abstract

Background: Olfactory disorders (ODs) are reported to be an early non-motor sign before the onset of deterioration in neurodegenerative diseases (NDs) such as Alzheimer’s and Parkinson’s. This systematic revision aims to review the current literature and the value of subjective olfactometry (SO) in the early diagnosis of cognitive decline and NDs. Methods: A systematic literature review was conducted following the PRISMA framework. Four different authors reviewed six different databases. The main variables analyzed were olfactory function and cognitive status. The quality of results was evaluated using the Oxford Centre of Evidence-based Medicine Levels. Results: Twenty-one cross-sectional and cohort studies and six meta-analyses were included. Most of them found an association between ODs and NDs. A prevalence of ODs greater than 80% was shown in Parkinson’s disease, proportional to the severity of symptoms. In Alzheimer’s, ODs were associated with early diagnosis and prognosis. All SO tests employed in the literature showed enough predictive value to correlate with early stages of cognitive decline. Conclusions: SO should be considered a pivotal tool when diagnosing NDs due to their association with early symptoms and prognosis. However, in the current literature, no firm consensus exists on the optimal SO tests and protocols that should be applied to the study of NDs, which prevents the interpretability and comparability of results among studies.

## 1. Introduction

Olfaction entails the sensory reception and cognitive processing of environmental odorants [1]. The diagnosis and treatment of olfactory disorders (ODs) still face important challenges due to the intricate nature of olfactory processes. ODs have increasingly gained the attention of researchers, evidenced by a growing number of works published in the last few years [2]. Nevertheless, with olfaction playing a fundamental role in many daily activities, such as nutrition, socio-affective interactions, and emotional processing, it remains a significant issue worthy of further research [3].

Recent advances in the understanding of the olfactory signal pathways at the primary cortex and its relationship with other brain areas have evidenced the role of ODs as early biomarkers for sinonasal diseases as well as for other cognitive-related disorders such as neurodegenerative diseases (NDs) [4]. Olfactory ability is known to exhibit an inverse correlation with age, with a prevalence of disorders rising from 18% at 60 years to 80% at 80 years of age in the general population [5]. Additionally, the early onset of ODs, particularly in odor recognition and identification, has been associated with cognitive impairment and NDs, with Alzheimer’s (AD) and Parkinson’s (PD) being the diseases in which these disorders are most prevalent [6,7,8]. The accumulation of peptides and structural changes within the limbic system and frontotemporal cortex reported in such neurological disorders have been related to a wide spectrum of dysfunctions including cognitive, sensory, autonomic, and motor domains [9]. Notably, olfactory symptoms have been reported to appear some years before the manifestation of other motor or cognitive symptoms [10]. This phenomenon has been attributed to an earlier pathological aggregation of proteins (e.g., alpha-synuclein aggregates) in olfactory regions [11]. Thus, ODs have been reported to be promising biomarkers for the early detection of NDs, including initial phases of mild cognitive impairment (MCI) [12,13].

Nowadays, the main technique employed for the investigation of ODs is based on subjective smell tests, due to their easy implementation, reproducibility, and relative cost-effectiveness [14]. In addition, the COVID-19 pandemic has promoted the development and validation of novel diagnostic tests, extending their applicability to diverse medical domains [2,15,16]. Consequently, recent investigations have highlighted the efficacy of olfactory assessment tools in facilitating the early detection and prognosis of NDs [17].

The aim of this study is to conduct a systematic review to evaluate the current literature and predictive value of subjective olfactometry (SO) in the early diagnosis of cognitive decline and NDs. This study aims to identify the advantages associated with the early diagnosis of ODs through standardized subjective olfactometry tests. These assessments aim to serve as predictive biomarkers for both the diagnosis and prognosis of NDs.

## 2. Materials and Methods

This systematic review has been conducted following the Preferred Reporting Items for Systematic Reviews and Meta-Analyses (PRISMA) guidelines (Supplementary Table S1) [18]. No review protocol was registered for this study.

### 2.1. Research Question

We aimed to answer the following research question: What is the role of subjective olfactometry in the early diagnosis of cognitive impairment and NDs?

### 2.2. Search Strategy

The search strategy was designed using the PICOTs framework [18]:

- Participants: Adult patients with cognitive impairment or NDs, with or without olfactory disorders assessed through subjective olfactometry.
- Intervention: Eligible interventions included standardized olfactometry tests as primary assessment tools.
- Comparators: Healthy adults with no diagnosis of an olfactory disorder or cognitive impairment.
- Outcomes: Diagnostic accuracy of subjective olfactometry tests for the early identification of cognitive impairment or NDs, and prognostic value of these tools in predicting the progression of NDs.
- r: Research works published in the last six years (from 2018 to 2023).

According to PRISMA recommendations, a search was conducted in the following databases: PubMed, The Cochrane Library for Cochrane Reviews, Embase via Elsevier, Web of Science, and Scopus from January 2018 to January 2024. The search strategy was performed using medical subject headings (MeSH) terms: (“olfaction” OR “olfactory disorder” [Title/Abstract] OR “olfactory disturbance” [Title/Abstract] OR “olfactory function” [Title/Abstract] OR “olfactory dysfunction” [Title/Abstract] OR “olfactory impairment” [Title/Abstract] OR “olfactory loss” [Title/Abstract] OR “smell” OR “smell impairment” [Title/Abstract] OR “chemosensory dysfunction” [Title/Abstract] OR “olfactometry” [Title/Abstract] OR “smell test” [Title/Abstract]) AND (“cognitive deficits” [Title/Abstract] OR “cognitive function” [Title/Abstract] OR “cognitive impairment” [Title/Abstract] OR “dementia” [Title/Abstract] OR “neurodegenerative” [Title/Abstract] OR “neurodegenerative diseases” [Title/Abstract] OR “Alzheimer” [Title/Abstract] OR “Parkinson” [Title/Abstract]). In order to double-check the database search, we manually checked the reference lists of the studies included and performed both a backward and forward citation analysis.

### 2.3. Eligibility Criteria

Inclusion criteria were: studies published in English or in Spanish, including prospective and retrospective cohort studies, case-control studies, randomized controlled trials (RCTs), longitudinal studies, and meta-analyses published in peer-reviewed journals, from January 2018 until January 2024. No specific criteria were considered for sinonasal diseases in this review.

Exclusion criteria were: studies that did not fulfill language criteria or were developed in animal specimens. Reviews, editorials, and commentaries, as well as studies with no clear relevance to the research question addressed (i.e., the role of subjective olfactometry in the early diagnosis of cognitive impairment), were excluded. Additionally, studies that did not report outcome measures for olfaction and/or cognitive status were also excluded.

### 2.4. Study and Variable Extraction

Screening by title and abstract was conducted by two authors (L.R.-C., D.M.-J.). After title and abstract screening and discarding, full texts were retrieved for the remaining articles. The same authors reviewed the full texts against the inclusion criteria. Discrepancies were solved by consensus.

Data extraction was conducted by four authors (L.R.-C., D.M.-J., B.V.-G, M.A.C.-L.). Extracted variables included: sample size, age, gender, olfactory outcome, and cognitive status; the latter two were measured through validated smell tests and clinical scales, respectively.

### 2.5. Assessment of Study Quality and Risk of Bias

The quality of the studies selected for the systematic review was assessed using the Oxford Centre for Evidence-Based Medicine Levels of Evidence [19]. The quality of the meta-analyses reviewed was measured based on an I^2^ index greater than 25% [20,21], which is interpreted as the percentage of the total variability in a set of effect sizes due to true heterogeneity. The risk of bias for quasi-experimental and cohort studies was assessed using the quality assessment of case series studies checklist from the National Institute for Health and Clinical Excellence [22].

### 2.6. Descriptive and Qualitative Analysis

A qualitative analysis was assessed by discussing the value and relevance of the articles included in the systematic revision. Four authors (L.R.-C., D.M.-J., B.V.-G., M.A.C.-L.) independently performed the evaluation with discrepancies being solved by consensus and reflected in the discussion section.

In our review analysis, studies included assessed olfaction outcomes through smell test scores. The diagnosis of olfactory disorders is made based on the number of correct responses in these psychophysical olfactory tests, with lower values being associated with worse olfaction outcomes. The studies included in the revision followed the classification of ODs reported by Patel et al. [2]. Normosmia is defined as the normal and healthy state of olfaction, based on data extrapolated from the olfactory capabilities of healthy individuals aged 16 to 35 [1]. Hyposmia (or microsmia) is defined as a partial reduction in the sense of smell, which can be further categorized into mild, moderate, or severe [23]. Finally, anosmia is defined as the absence of olfaction, together with the inability to detect and precisely identify odors [24]. Olfactory tests assess the ability to detect, identify, and differentiate odors. The variables typically measured in these tests include: (i) detection (i.e., the ability to perceive an odor), (ii) identification (ability to select the odorant from a list of options), (iii) discrimination (ability to differentiate odors and determine whether they are similar or different from each other), (iv) threshold (the lowest odor concentration a person can detect), (v) olfactory memory or recognition of previously presented odors, and (vi) odor tolerance (ability to withstand or resist exposure to a particular odor without experiencing annoyance, discomfort, or adverse reactions). There exist several olfactory tests, and the variables measured vary depending on the specific test used and the purpose of the assessment. Further information about the methodological differences across smell tests used in the reviewed studies can be found in Supplementary Table S2.

Cognitive impairment was assessed through validated scales and questionnaires, as specifically measured and interpreted in the respective studies analyzed. Most of these studies included a global cognitive screening evaluation based on the Montreal Cognitive Assessment (MoCA) or the Mini-Mental State Examination (MMSE). While some studies used well-established neuropsychological batteries such as the Alzheimer’s Disease Assessment Scale-(Japanese version) Cognitive Subscale (ADAS-Jcog) [25], the Seoul Neuropsychological Screening Battery [26], or the Scales for Outcomes in Parkinson’s Disease-Cognition (SCOPA-Cog) [27], other studies created their own battery by compiling different scales and tests to evaluate different cognitive domains that are known to be deteriorated in NDs, including attention, language, memory, executive functions, working memory, visuospatial abilities, and others. Supplementary Tables S3 and S4 offer detailed information about the different cognitive scales and tests used in the reviewed studies. Other complementary tests reported in these studies, such as neuroimaging tests, are also reported.

## 3. Results

**Figure 1:**
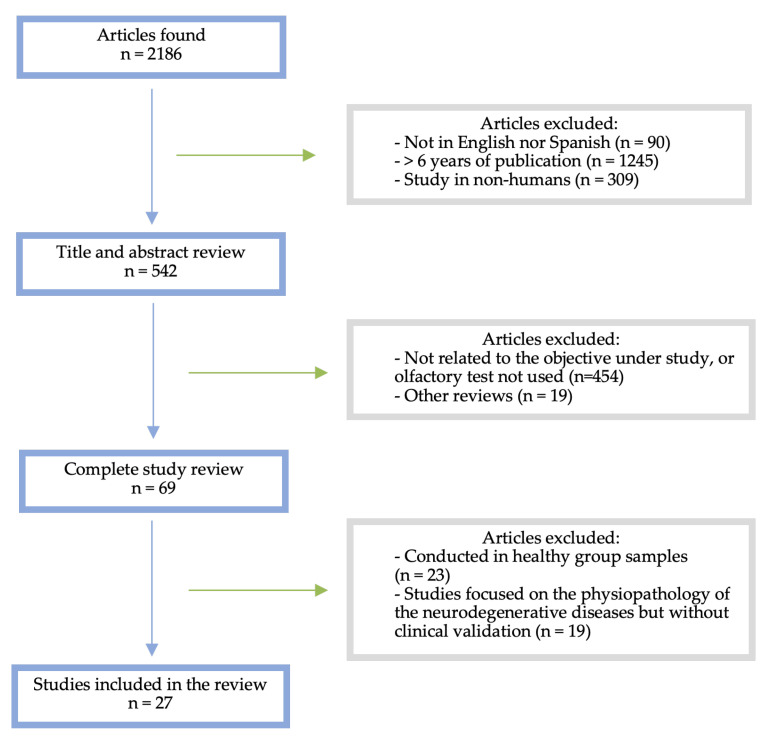
PRISMA flow diagram.

The bibliographic search was performed from February 2023 to January 2024, which allowed us to identify 2186 potentially relevant studies. After removing duplicates and applying the inclusion and exclusion criteria, twenty-seven articles were included following a PRISMA flow diagram (see Figure 1). Twenty-seven articles relevant to the subject under study were included: twenty-one cross-sectional and/or cohort studies and six meta-analyses.

Table 1 summarizes some relevant variables from the reviewed studies, including the sample size and mean and range of age of the participants. In Table 1, studies are grouped according to different the diseases assessed. Specifically, thirteen studies assessed PD, five assessed AD and mild/moderate cognitive impairment (MCI), two studies assessed multiple sclerosis (MS), and one study addressed amyotrophic lateral sclerosis (ALS).

### 3.1. Cross-Sectional and Cohort Studies

Table 2 describes the main results derived from the cross-sectional and cohort studies reviewed. The variables reported are: the reference of the study, the quality of the study according to the metrics provided by the Oxford Centre for Evidence-Based Medicine Levels of Evidence, the sample size of patients and healthy controls, the mean age, the proportion of gender, the olfactometry tests, and other tests used to diagnose and characterize the NDs. Finally, the main outcomes and limitations are also listed. A description of the main results derived from each reviewed study is provided below.

Among the groups that studied olfactory disorders in PD, Masala et al. [28] reported a prevalence of 99% of ODs in the group of patients analyzed, involving odor identification, threshold, and discrimination, as well as a correlation between smell identification and motor symptoms’ severity. Jalali et al. [29] found a prevalence of 96.8% of smell disorders in PD, with more olfactory impairment in patients with tremor dominance (TDPD). Roos et al. [30] reported a correlation between ODs and autonomic and sleep impairment, depression, and anxiety in PD, also proportional to the severity of symptoms. In addition, they also observed a relationship between worse olfaction outcomes and a higher number of nigrostriatal dopaminergic neurons damaged in the caudate nucleus and the putamen. In Yoo et al. [26], a greater proportion of patients with hyposmia and olfactive anosognosia (OA)—the latter defined as the lack of self-acknowledgment of smell impairment—presented basal mild cognitive impairment (MCI) and dementia during the follow-up, in addition to a more accelerated clinical worsening of executive and global cognitive functions in comparison with normosmic patients or those without OA. Lee et al. [31] found that normosmic patients improved their axial symptoms more than those with ODs. Moreover, the group of patients with ODs developed a freeze of gait more frequently during their follow-up. In the study by Elhassanien et al. [32], a significant deterioration of all olfactory domains and a decrease in olfactory bulbs were observed in patients with TDPD in comparison with those with PD and healthy controls. Trentin et al. [33] reported lower threshold, discrimination, and identification index (TDI) scores in PD patients, suggesting the greater predictive power of this index measure compared with smell identification and discrimination.

Other authors have also analyzed ODs in different variants of PD. For example, Saunders-Pullman et al. [34] evaluated a group of patients with the LRRK2 variant against a group with idiopathic PD, concluding that the latter presented worse olfaction outcomes. Nabizadeh et al. [35] investigated the relationship between ODs and other motor and non-motor symptoms in three subtypes of PD: undetermined, TDPD, and postural instability with gait deterioration (PIGD). They only found a correlation between ODs and motor and non-motor impairment in the TDPD group. Stewart et al. [36] reported worse smell scores and higher MRI-based impairment in patients with MCI and PD compared with those with normal cognition, in proportion to the severity of symptoms.

Other studies have not found any relationship between ODs and cognitive impairment in PD patients. For example, Camargo et al. [27] did not find any correlation between ODs and cognitive impairment in PD; however, they did find a correlation with attention deficits. Fujio et al. [37] found no relationship between ODs and cognitive impairment during three years of follow-up.

Regarding AD, Yoshii et al. [25] found a significant correlation between ODs and cognitive impairment, especially in domains and tasks involving word memory, orientation, and ideational praxis. Also, ODs correlated with temporal medial lobe atrophy. Lian et al. [38] reported a significant global cognitive, attention, speech, and visuospatial function impairment in patients with ODs, as well as a significant reduction in hippocampal and amygdala volume, among other structures. Doordujin et al. [39] described lower smell scores in AD and MCI patients, especially in smell identification and discrimination. In Wang et al. [12], smell identification impairment was seen in subjective cognitive impairment (SCI) and worsened in MCI and AD. They also found a correlation between smell identification and cognitive deterioration in AD. Thomas et al. [13] described more severe ODs in patients who presented MCI with Lewy bodies.

When assessing MS and ALS, Da Silva et al. [40] described greater symptom severity and shorter survival times in patients with ODs after a 10-year follow-up. They also found a greater death risk in these patients. Duz et al. [41] reported significant ODs in relapsing-remitting MS (RRMS). Threshold detection was significantly impaired in radiologically isolated syndrome (RIS). Both groups showed cognitive impairment when smell was also impaired. Finally, a strong correlation was described between worse olfactory threshold detection and the early inflammatory stage of the disease. In Masuda et al. [42], the smell test score was significantly lower in ALS patients and correlated with frontotemporal cognitive dysfunction.

Notably, the majority of these studies fall within 3b and 4 evidence levels. Supplementary Table S5 collects the risk of bias for these studies. The inclusion criteria followed in our review, specifically designed to analyze the currently available research regarding the usability and accuracy of olfactometries in ND diagnosis, show that studies with higher levels of evidence are still limited.

**Table 2:** Summary of the cross-sectional and cohort studies included in the review.

Authors	Study Design and Level of Evidence	Follow-Up (Years)	Sample Size	Age (Mean ± SD)	Gender (M/W)	Main ND Disease Tests	Smell Test	Sinonasal Diseases Considered	Prevalence of Olfactory Disorders	Main Outcomes
Camargo et al., Brazil (2018) [27]	Cross-sectional 4	N/A	PD: 42 Controls: 38	PD: 70.7 ± 10.7 C: 69.2 ± 6.5	PD: 62/38 C: 53/47	H and Y Scale UPDRS-III SCOPA-Cog MMSE MDS Criteria	SST-12	No	PD: 95% C: NR	No correlation between cognitive impairment and SST-12. Correlation between lower SST-12 score and attention loss measured through SCOPA-Cog.
Masala et al., Italy (2018) [28]	Cross-sectional 4	N/A	PD: 96 Controls: 51	PD: 67.8 ± 8.2 C: 65.1 ± 11.8	PD: 59/37 C: 25/26	Gelb criteria UKPDSBB H and Y Scale UPDRS-III MoCA	SST	Yes	PD: 99% C: 33%	Negative correlation between motor symptoms’ severity, SI and TDI. Negative correlation between apathy, ST, SD, SI and TDI.
Jalali et al., Iran (2019) [29]	Cross-sectional 4	N/A	PD: 104	PD: 64.1 ± 5.7	PD: 66/38	MDS Criteria H and Y Scale	Iran-SIT	Yes	PD: 97%	Lower Iran-SIT scores correlated with advanced age (>60 y). Negative correlation between Iran-SIT and H and Y scale. Differences in SI across PD subtypes: Lower Iran-SIT scores in TDPD than in PIGD or LOPD.
Yoo et al., Republic of Korea (2019) [26]	Retrospective cohort 3b	5	PD: 77 Normosmic: 15; Hyposmic without anosnognosia (AO−): 40; Hyposmic with anosognosia (AO+): 22	Normosmic: NR Hyposmic AO−: NR Hyposmic AO+: NR	Normosmic: 6/9 Hyposmic AO−: 23/17 Hyposmic AO+: 11/11	UKPDSBB UPDRS-III RBD Screening Questionnaire BDI MMSE	CCSIT	Yes	PD: 80.5%	Higher proportion of MCI at baseline in patients with hyposmia and AO. Greater tendency to dementia and more rapid decline in MMSE in patients with hyposmia. Greater conversion to dementia in the group of hyposmia and AO+.
Roos et al., The Nether- lands [30]	Cross-sectional 4	N/A	PD: 295, within them 155 with DaT-SPECT	PD: 65.3 ± 10.4 DaT-SPECT: 65.4 ± 10.7	PD: 179/116 DaT-SPECT: 95/60	UKPDSBB H and Y Scale UPDRS-III MMSE SCOPA BDI/BAI DaT-SPECT	UPSIT	N/R	PD: 83%	Lower UPSIT scores correlated with sleep disorders, depression and anxiety. Association between smell impairment and severity of motor symptoms. UPSIT scores are directly proportional to dopaminergic neurons loss at the caudal nucleus and putamen as measured by DaT-SPECT
Yoshii et al., Japan (2019) [25]	Cross-sectional 4	N/A	AD: 55 MCI: 27	AD: 80 ± 7 MCI: 76 ±10	AD: 21/34 MCI: 8/19	DSM-IV criteria NINCDS NIA-AA ADAS-Jcog MRI	OSIT-J	Yes	N/R	Significant differences between OSIT-J and AD and MCI. Positive correlation between OSIT-J and ADAS-Jcog and OSIT-J and brain atrophy. Association between OSIT-J scores and medial temporal lobe atrophy (hippocampus and parahippocampal region).
Lian et al., China (2019) [38]	Cross-sectional 4	N/A	AD: 60 (30 with ODs, 30 without)	ODs: 66.4 ± 11.7 No ODs: 65.3 ± 10.0	SI: 33/67 No SI: 43/57	NIA-AA MMSE AVLT BNT ADL MRI	SST	Yes	N/A	Positive correlation between ST and SI and global cognition. Significant impairment of cognitive function, memory, attention, speech and visuospatial capacities if olfactory dysfunction. Significantly smaller hippocampal and amygdala volume if olfactory dysfunction. Thinner entorhinal, inferior temporal, middle temporal and fusiform cortices. Positive correlation between MMSE and TDI.
Doorduijn et al., The Netherlands (2020) [39]	Cross-sectional 4	N/A	AD: 30 MCI: 22 Controls: 40	AD: 62.5 ± 6.8 MCI: 69.8 ± 7.2 C: 69.5 ± 9.4	AD: 18/22 MCI: 16/6 C: 14/16	NIA-AA MMSE TMT A	SST	No	AD: 77% MCI: 68% C: 38%	Lower SD and SI in AD and MCI. No difference in ST between the 3 groups. Association between low TDI, SI and SD and memory impairment.
Da Silva et al., Portugal (2020) [40]	Retrospective cohort 3b	10	MS: 149	MS: 41 (35–50)	MS: 47/102	EDSS MSSS ARMSS MMSE	B-SIT	Yes	RRMS: 4% SPMS: 56% PPMS: 12%	Smell dysfunction does not predict the switch from RRMS to progressive MS. More severe symptoms in the follow-up of progressive MS and smell dysfunction. Greater death HR if B-SIT impaired.
Fujio et al., Japan (2020) [37]	Prospective cohort 4	3	PD: 56	PD: 67.8	PD: 27/29	MMSE	JSO OE	Yes	PD: 88%	No correlation between OE and lower MMSE between the 1st and last evaluation.
Lee et al., South Korea (2021) [31]	Retrospective cohort 3b	1	PD: 108 (Normosmia: 29, Hyposmia: 79)	N-PD: 58.9 ± 10.6 H-PD: 66.2 ± 9.1	N-PD: 19/10 /> H-PD: 43/36	MDS criteria UPDRS MMSE MoCA	KSST	Yes	PD: 73%	Significant improvement of motor functions in N-PD with treatment rather than in H-PD (axial symptoms). Higher development of freezing of gait in H-PD.
Masuda et al., Japan (2021) [42]	Cross-sectional 4	N/A	ALS: 30 Controls: 53	ALS: 67.3 ± 8.2 C: 68 ± 6.9	ALS: 28/25 C: 20/10	El Escorial criteria ALSFRS-R MMSE FAB ADAS-Jcog BDI MRI	OSIT-J	Yes	N/R	Significantly lower OSIT-J in ALS. Positive correlation between OSIT-J and frontotemporal cognitive impairment, bilateral medial orbital cortex and right hippocampus atrophy in ALS.
Duz et al., Turkey (2021) [41]	Cross-sectional 4	N/A	RRMS: 10 RIS: 10 Controls: 10	RRMS: 37 ± 9.5 RIS: 33.2 ± 7.5 C: 33.4 ± 3.5	RRMS: 2/8 RIS: 3/7 C: 3/7	MMSE BDI EDSS	SST	Yes	N/R	Significative smell dysfunction in RRMS. ST impairment in RIS. Cognitive impairment in RRMS and RIS: attention, memory and executive functions. Association between ST decrease and early inflammatory stage in MS. SI and SD impairment if neurodegeneration.
Elhassa-nien et al., Egypt (2021) [32]	Cross-sectional 4	N/A	TDPD: 22 ET: 36 Controls: 24	TDPD: 57.7 ± 3.5 ET: 62.6 ± 4.6 C: 62.0 ± 6.7	TDPD: 14/8 ET: 20/16 C: 16/8	MDS criteria UPDRS-III MRI	SST	Yes	TDPD: 100% ET: 75% C: 29%	Significant ST, SI, SD and TDI decrease in TDPD in comparison with ET and C; and in ET in comparison with C. Significant olfactory bulb volume decreases in TDPD. Negative correlation between PD duration and smell dysfunction.
Wang et al., China (2021) [12]	Cross-sectional 4	N/A	SCI.: 84 MCI: 129 AD: 52 Controls: 35	SCI: 67 ± 5.6 MCI 67.8 ± 8.6 AD: 71.2 ± 10.3 C: 67.5 ± 5.3	SCI: 32/52 MCI: 41/88 AD: 23/29 C: 18/17	NINCDS Peterson criteria MMSE AVTL BNT	SST-16	No	SCI: 54% MCI: 65% AD: 96% C: 23%	Worse SI is associated with worse cognition in AD. Strong positive correlation between SI and memory.
Trentin et al., Brazil (2022) [33]	Cross-sectional 4	N/A	PD: 27 Controls: 17	PD: 65.6 ± 9.7 C: 61.4 ± 7.4	PD: 11/16 C: 3/14	MDS Criteria MoCA	SST	Yes	PD: 100% C: 53%	Significantly worse ST, SI, SD and TDI in PD. Positive correlation between cognition and TDI. Superiority of TDI vs its subtests.
Saunders-Pullman et al., USA (2022) [34]	Prospective cohort 4	3.4	PD LRRK2: 162 IPD: 198	PD LRRK2: 67.6 ± 9.5 IPD: 65.4 ± 10.5	PD LRRK2: 54/46 IPD: 63/37	UKPDSBB UPDRS MoCA GDS	UPSIT	N/R	PD LRRK2: 56% IPD: 85%	PD LRRK2: worse UPSIT were significantly younger. Correlation between worse UPSIT and motor impairment progression. Greater deterioration if worse UPSIT in IPD.
Thomas et al., UK (2022) [13]	Cross-sectional 4	N/A	Probable MCI-Lewy Bodies:38 Possible MCI-LB: 19 MCI-AD:33 Controls: 32	Prob. MCI-LB: 74.1 ± 6.6 Pos. MCI-LB: 73 ± 7.3 MCI-AD: 74.6 ± 7.5 C: 73.9 ± 7.2	Prob. MCI-LB: 33/5 Pos. MCI-LB: 10/9 MCI-AD: 16/17 C: 23/9	MDS Criteria NIA-AA UPDRS-III GDS	SST-16	No	Prob. MCI-LB: 84% Pos. MCI-LB: 74% MCI-AD: 70% C: 34%	Significant correlation between cognition and SST-16, not with motor dysfunction. No correlation between SST-16 and subjective assessment of olfaction. ≤7 SST-16 cutoff: differentiate MCI-LB from MCI-AD.
Almeida et al., Brazil (2022) [17]	Cross-sectional 4	N/A	PD: 20 Controls: 9	PD: 49.8 ± 5.0 C: 60.8 ± 9.6	PD: 13/7 C: 6/3	UKPDSBB H and Y Scale UPDRS-III ADL MMSE DaT-SPECT TCS	SST-16	Yes	PD: 65% C: 0%	Positive correlation between SST-16 and SPECT, negative correlation between SST-16 and TCS. SST-16 + TCS assessment compares to SPECT (gold standard) to confirm PD.
Nabizadeh et al., Iran (2022) [35]	Cross-sectional and prospective cohort 3b	4	PD: 487 Controls: 197	PD: 61.7 ± 9.7 C: 61.4 ± 11.0	PD: 335/152 C: 131/66	MDS Criteria H and Y Scale UPDRS ADL MoCA GDS SCOPA-AUT	UPSIT	Yes	N/R	Greater smell impairment in PD than in controls. Positive correlation between UPSIT and H and Y stage, motor and non-motor impairment in tremor-dominant patients. No correlation with other subgroups of PD.
Stewart et al., Canada (2023) [36]	Cross-sectional 4	N/A	PD-MCI: 12 PD-Normal cognition: 21	PD-MCI: 63.5 ± 7.28 PD-NC: 61.4 ± 5.95	PD-MCI: 6/6 PD-NC: 13/8	H and Y Scale MDS Criteria TMT MRI (DTI)	UPSIT	No	N/R	Better UPSIT scores and lower changes in MRI-DTI measures in PD-NC. Positive correlation between the severity of smell dysfunction and cognitive impairment.

ADAS-Jcog: Alzheimer’s Disease Assessment Scale—Cognitive Subscale Japanese Version, ADL: Activities Of Daily Living, ALS: Amyotrophic Lateral Sclerosis, AO: Anosognosia, ALSFRS-R: Amyotrophic Lateral Sclerosis Functional Rating Scale-Revised, ARMSS: Age-Related Multiple Sclerosis Severity, AVLT: Auditory Verbal Learning Test, BAI: Beck Anxiety Inventory, BDI: Beck Depression Inventory, BNT: Test Boston Naming, B-SIT: Brief-Smell Identification Test, CCSIT: Cross-Cultural Smell Identification Test, Dat-SPECT: Dopamine Transporter Single-Photon Emission Computed Tomography, DSM: Diagnostic and Statistical Manual Of Mental Disorders, DTI: Diffusion Tensor Image, EDSS: Extended Disability Status Scale, ET: Essential Tremor, FAB: Frontal Assessment Battery, GDS: Geriatric Depression Scale, H and Y: Hoehn and Yahr, HR: Hazard Ratio, IPD: Idiopathic Parkinson’s Disease, Iran-SIT: Iran Stick Identification Test, JSO: Jet Stream Olfactometry, KSST: Sniffin’ Stick Test Korean Version, LOPD: Late-Onset Parkinson’s Disease, MCI: Mild Cognitive Impairment, MDS: Movement Disorder Society, MMSE: Mini-Mental State Examination, Moca: Montreal Cognitive Assessment, MRI: Magnetic Resonance Imaging, MSSS: Multiple Sclerosis Severity Scale, N/A: Not Applicable, ND: Neurodegenerative Disease, NIA-AA: National Institute On Aging-Alzheimer’s Association, NINCDS: National Institute Of Neurological and Communicative Disorders and Stroke, N/R: Not Reported, OD: Olfactory Disorder, OE: Open Essence, OSIT-J: Odor Stick Identification Test For Japanese, PET: Positron Emission Tomography, PIGD: Postural Instability and Gait Difficulty, PPMS: Primary Progressive Multiple Sclerosis, RBD: Rapid Eye Movement Sleep Behavior Disorder, RIS: Radiologic Isolated Syndrome, RRMS: Relapsing-Remitting Multiple Sclerosis, SCI: Subjective Cognitive Impairment, SCOPA-AUT: Scales For Outcomes In Parkinson’s Disease-Autonomic Dysfunction, SCOPA-Cog: Scales For Outcomes In Parkinson’s Disease-Cognition, SD: Smell Discrimination; SI: Smell Identification, SPMS: Secondary Progressive Multiple Sclerosis, SST: Sniffin’ Sticks Test, ST: Smell Threshold, TCS: Transcranial Sonography, TDI: Threshold Discrimination and Identification Index, TDPD: Tremor Dominant Parkinson’s Disease, TMT: Trail Making Test, TMT A: Trail Making Test Part A, UKPDSBB: Parkinson’s Disease Society Brain Bank, UPDRS: Unified Parkinson’s Disease Rating Scale, UPSIT: University Of Pennsylvania Smell Identification Test.

### 3.2. Meta-Analyses

The meta-analyses included in this systematic revision reported a correlation between olfactory disorders and neurodegenerative diseases in almost all studies included. The University of Pennsylvania Smell Identification Test (UPSIT) [43] was the test most frequently used for the study of olfaction in patients with dementia. The results and quality of meta-analyses included are listed in detail in Table 3 and are described below.

Sui et al. [44] studied the association between hyposmia and PD in seven research papers analyzed. A significant relationship was observed between hyposmia and a greater risk of PD. Alonso et al. [45] reported smell identification impairment in PD patients in comparison with controls in 104 studies reviewed, irrespective of short- or long-term PD. Janssen Daalen et al. [46] found that ODs might suggest PD diagnosis in populations with a high proportion of elderly women and in patients with REM sleep behavior disorder, according to eight studies assessed. Kotecha et al. [47] described a significant smell identification impairment in AD and MCI patients in comparison with controls in 10 studies reviewed. In Jung et al. [48], the authors showed a greater rate of ODs in patients with Alzheimer’s compared with those with MCI, especially in the subdomain of smell identification, according to 12 research papers assessed. Lastly, Jobin et al. [49] reviewed five studies and reported mild impairment in smell identification in patients with SCI in comparison with controls.

**Table 3:** Summary of the meta-analysis included in the review.

Authors	Neurodegenerative Disease	Articles Included	Exclusion Criteria	Smell Test Assessed	Results	I^2^
Kotecha et al., UK (2018) [47]	Alzheimer’s disease (AD) Mild cognitive impairment (MCI)	10	Insufficient characterization of AD and MCI sample, no control group, incorrect olfactory measurement/methodology, non-extractable raw data, non-randomized and non-controlled trials.	UPSIT-10 (4) SSIT-16 (3) UPSIT-12 (1) UPSIT-20 (1) SSIT (1) SSIT-12 (1) SSDT-16 (1) B-SIT-12 (1)	Significant SI differences between AD patients and controls. Significant SI differences between MCI patients and controls.	AD: 75% MCI: 61%
Sui et al., China (2019) [44]	Parkinson’s disease (PD)	7	If published as case-control, cross-section, case report, review, conference abstract, comment or letter, reported only risk estimates, if 95% CIs were not reported, non-sufficient data to calculate risk estimates, duplicate populations, non-English publications, if not published.	SST (2) BSIT (2) UPSIT (1) SDOIT (1) Not specified (1)	Correlation between hyposmia and increased risk of PD. Greater risk of PD development if hyposmia is presented before the diagnosis in comparison with healthy controls.	51.6%
Jung et al., South Korea (2019) [48]	Alzheimer’s disease (AD) Mild cognitive impairment (MCI)	12	Use of animals, comorbid neurological conditions that affect olfactory function, reviews or symposium papers.	UPSIT (6) SST (2) BSIT (1) CA-SIT (1) CCSIT (1) 16 common odors (1)	Greater olfactory dysfunction in AD than in MCI patients. Greater SI compared with ST or SD.	45.5%
Janssen Daalen et al., The Netherlands (2021) [46]	Parkinson’s disease (PD)	8	Cross-sectional studies.	UPSIT (4) BSIT (3) SST-16 (2) SST-12 (1)	Olfactory dysfunction may indicate PD diagnosis in populations with a higher proportion of older women and in patients with REM sleep behavior disorder.	70.7%
Alonso et al., Brazil (2021) [45]	Parkinson’s disease (PD)	104	Not published in a peer-reviewed journal, published as editorials, letters, comments, review articles, longitudinal studies, or single-case studies.	UPSIT (45) SST-48 (18) SST-16 (16) B-SIT (13) OSIT-S (7) SST-12 (6) Open Essence (2)	Significant olfactory impairment in PD in comparison with controls, regardless of the test used. Lower heterogeneity (I^2^) if UPSIT is used compared to other tests. Long term PD patients did not have a significantly different olfactory dysfunction than patients recently diagnosed.	70%
Jobin et al., Canada (2021) [49]	Subjective cognitive impairment (SCI)	5	<50 y/o, if cognitive impairment, psychiatric diagnosis or neurological condition were present.	SSIT (2) UPSIT (1) CC-SIT (1) OPID (1)	Mild SI worsening detectable in SCI patients compared to controls.	30%

BSIT: Brief-Smell Identification Test, CA-SIT: Culturally Adapted Smell Identification Test, CC-SIT: Cross-Cultural Smell Identification Test, I^2^: Heterogeneity, OPID: Odor Percept Identification, REM: Rapid Eye Movement, SD: Smell Discrimination, SDOIT: San Diego Odor Identification Test, SI: Smell Identification, SSDT: Sniffin’ Sticks Discrimination Test, SSIT: Sniffin’ Sticks Identification Test, ST: Smell Threshold, UPSIT: University of Pennsylvania Smell Identification Test.

## 4. Discussion

As reviewed in this study, olfactory function can be an early or pre-clinical marker of neurodegenerative disease. Growing evidence also suggests that odor identification deficits can be a marker for cognitive outcomes. The interest in the study of olfaction lies not only in the ability to help diagnose patients with cognitive impairment in its early stages but also in its contribution to a better characterization of the disease, as well as the clinical possibilities that olfactory training may provide in terms of ND prevention, control, and rehabilitation.

This systematic review analyzes the predictive role of subjective olfactometry in the early detection and prognosis of NDs. We have retrieved twenty-one original articles and six meta-analyses published in the last six years, providing information about smell impairment in four different neurological diseases (PD, AD, MS, ALS). The main results derived from this review are discussed below.

### 4.1. Prevalence and Predictive Value of Olfactory Disorders in NDs

In PD, most of the studies reviewed described greater smell dysfunction in ND patients than in controls, thus suggesting olfactory impairment as a disease’s risk factor [17,26,28,29,30,31,32,33,34,35,36,44,45,46]. The mean prevalence of olfactory impairment in PD was 83.8 ± 14.5 across eleven studies, with some of them reporting a negative correlation between olfactometry outcomes and disease impairment [28,30]. Such impairment was observed in cognition, attention, and motor symptoms, as well as in function and structure assessed with DaT-SPECT. PD patients also exhibited a more pronounced atrophy of olfactory pathway structures in neuroimages than healthy controls [25,30,32]. Janssen et al. suggested that, in populations with a higher proportion of older women and patients with REM sleep behavior disorder, olfaction impairments are indicators of PD diagnosis [46]. Better response to PD treatment has also been observed, especially in axial symptoms, in normosmic compared to hyposmic patients [31]. In addition, different grades of severity of ODs were also found among different PD variants, with worse olfactory outcomes in the idiopathic and early-onset forms [34]. Other authors have also found worse olfactory function in patients with tremor-dominant PD [29,32]. Therefore, since olfaction deterioration may correlate with the characteristics, progression, and prognosis of the disease, the assessment of olfactory function may provide clinicians with useful information to be monitored when a PD diagnosis is suspected. However, the fact that other studies here-reviewed did not find an association between sense of smell and cognitive impairment [27,37] must also be taken into account. This was explained by the authors as possibly being due to a lack of specificity of the overall punctuation in the MMSE cognitive test related to PD function [37] and to a non-balanced study sample with a greater number of patients affected by long-term dementia [27].

Individuals with Alzheimer’s showed worse olfactory outcomes measured through different smell tests, compared with MCI patients and healthy controls [12,25,39]. Although in our review only five AD studies complied with inclusion criteria, all of them agreed to highlight a robust correlation between olfaction status and memory [12,13,25,38,39,47,48]. This was especially evident in patients who showed difficulties in odor discrimination, where memory plays a key role [38,48]. Pathological results in olfactometry also correlated with structural alterations in neuroimaging tests in patients with AD [25,38]. Additionally, two meta-analyses further supported the notion of increased smell dysfunction as AD progresses, particularly affecting smell identification [47,48]. Moreover, the association between cognition and olfaction remains true even when assessing sample groups without a definitive diagnosis of dementia at the baseline. Notably, individuals who initially show significant olfactory dysfunction tend to exhibit a higher likelihood of developing dementia, especially in AD [50]. Although only two studies involving MS and ALS met the inclusion criteria in our review, both of them underlined the deterioration and worse olfaction outcomes found in these patients [40,41,42].

### 4.2. Study of Olfactory Subdomains in NDs

The analysis of olfactory function and domains reveals some peculiarities in the diagnosis of NDs. From the results of this systematic review, the three olfactory domains were altered in PD [28,32,33], while in AD, the identification domain was the most affected, with increased severity of symptoms as the cognitive impairment progressed at different disease stages [12,13,25,38,39,47,48]. These published studies also reported greater olfactory dysfunction in AD compared to MCI patients [47,48]. In MS, detection thresholds were more impaired during the inflammatory phase, whereas identification and discrimination were mainly altered in the phase of neurodegeneration, thus possibly indicating the usefulness of olfactory function to help better characterize MS patients [41]. A correlation between ODs and higher mortality rates was also described for these patients [40]. Although only one study was included in our review for the analysis of ALS, this underlines the deterioration in smell identification and associates it with frontotemporal function impairment [42]. Changes in olfactory function, with peculiar characteristics in each ND, reinforce the need for smell assessment as part of ND diagnosis and management. However, available data are still insufficient and inconclusive, with further studies still needed in order to determine the exact role of olfactory function in NDs.

### 4.3. Subjective Olfactometries

Possibly driven by the increasing interest in olfaction research [14], there are several subjective olfactometries available today to evaluate the sense of smell (see Supplementary Table S2). Most of these tests only assess smell identification [43,51,52,53], with the SST being the only validated test addressing the three domains (identification, discrimination, and threshold), as well as the combined threshold–discrimination–identification (TDI) index [54]. Although the TDI index has been suggested to exhibit greater predictive value compared with their counterpart subdomains alone [32,33,38], the full version of the SST was only used in 6 out of the 21 papers reviewed (Table 2) [28,32,33,38,39,41]. The meta-analyses reviewed [44,45,46,47,48,49] also suggested that the SST may be the most suitable test when evaluating NDs as it offers more comprehensive information about these three different olfaction domains [12,13,17,39,40,43,44,45,48,49,50,54].

However, it must be noted that in addition to the fact that there is some divergence in the literature, with some studies reporting no differences in threshold or discrimination domains between cognitively impaired and healthy patients [38,39], a major limitation of extended tests such as the SST lies in the time required and the need for trained personnel, which may compromise its use as a screening clinical tool in dementia. Some authors have proposed the use of an easier and shortened version of this test (i.e., SST-12 and/or -16 items), for which scores can be equated to those derived from the extended version, demonstrating its usefulness in patients with NDs [55,56].

Moreover, the advantages of olfactometries—noninvasiveness, simplicity, and ease of use—make them affordable tools for the management of dementia. Future studies analyzing the predictive value and accuracy of these tests, together with the alterations of specific olfactory subdomains (i.e., threshold, discrimination, and identification) for each ND, could lay the groundwork for the definitive inclusion of olfactory testing in dementia screening.

### 4.4. Olfaction Outcomes in Relation to Neurocognitive Batteries Assessing Different Cognitive Domains

Although it is known that cognition plays an important role in olfaction, the cognitive domains involved in this process are still not accurately defined. Such discrepancy may be due to the methodological variability and heterogeneity of cognitive domains and tests evaluated in each study. In this review, cognitive evaluation ranged from general global cognitive screening tests to more comprehensive neuropsychological batteries addressing different cognitive domains, including attention, executive functions, language, word fluency, memory, and recognition, among others (see Supplementary Tables S3 and S4).

To assess global cognition, the MoCA and MMSE were the tests most commonly used in the reviewed studies, confirming the association between olfactory disorders and lower overall cognitive scores in PD and AD patients [30]. Other studies have used more comprehensive neuropsychological batteries to support the association of specific cognitive domains and olfactory disorders in neurodegenerative diseases. For example, SCOPA-Cog and SNSB, tested in PD patients, showed a correlation between olfactory disorders and attention, executive function [27], and visuospatial and frontal executive domains [26]. AD patients evaluated through ADAS-Jcog showed a correlation between olfactory function and memory and language domains [25]. In AD patients with olfactory disorders, impairments in verbal memory (i.e., AVLT and RAVLT) [39], language (i.e., BNT), and visuospatial ability (i.e., RCFT) [38] were identified. In addition, smell test score was associated, in ALS patients, with frontotemporal dysfunction (i.e., FAB), word fluency, memory, and recognition (i.e., ADAS-Jcog subtest) [42]. In MS patients, olfactory impairment was associated with verbal fluency and memory, attention, visuospatial abilities, and executive function (i.e., VMLT, visual reproduction WMS, digits span WMS, SWCT, BJLO, and BFRT) [41].

On the other hand, it is also important to mention that additional studies have found no association between olfactory disorders and cognitive screening tests in PD patients [41]. Although the authors explained that this lack of correlation was possibly due to the use of non-specific global cognition tests such as the MoCA and MMSE, other authors did not find an association between olfaction and specific cognitive domains when using a more enriched neuropsychological battery evaluating executive functions, working memory, visuospatial abilities, and speed-attention processing [41]. Therefore, the analysis of the specific relationship of ODs respective to different cognitive domains deserves more attention; the design and validation of novel comprehensive and interdisciplinary battery tests evaluating different cognitive capabilities and olfaction domains are still needed. This hopefully may help further our understanding of the specific brain pattern regions involved in cognitive and olfaction impairment. We believe that novel protocols evaluating both olfactory and cognitive function and subdomains are still needed to further understand their relationship and interdependence, as well as to better characterize the clinical profile of patients in each subset of NDs in relation to ODs [57].

### 4.5. Olfactory Training

Even though olfactory training is outside of the scope of this review, we must highlight that it has been reported to be an emergent tool for treating patients with NDs. Recent research indicates that olfactory training can lead to a decrease in depressive symptoms and an improvement in some cognitive functions, such as memory, attention, and speech [9], in patients with dementia. In addition, structural changes have been evaluated with neuroimaging tests in patients with MCI during olfactory training, showing an increase in the cortical thickness of the hippocampus as improved smell discrimination, without significant alterations in the dimensions of the olfactory bulb. Therefore, this intervention has been suggested to prevent hippocampal atrophy in these cases [58].

A recent review further supports the notion that olfactory stimuli can significantly impact self-related knowledge (e.g., understanding one’s identity, beliefs, traits, etc.). Olfactory cues have been found to improve access to autobiographical memories, particularly when triggered by specific odors, subsequently enhancing patients’ overall quality of life and well-being [59]. This effect can be attributed to the close connection between the olfactory bulb and the hippocampus—the brain region responsible for memory formation and consolidation. This highlights the intricate relationship between olfaction and involuntary memory retrieval, with no involvement of executive functions [60].

### 4.6. Underlying Basis of the Link between Olfactory Disorders and Cognitive Impairment

The findings derived from our review agree with other previous studies that have also suggested that olfactory dysfunction has a high predictive value for developing cognitive deficits [61,62]. However, the mechanisms by which olfactory function is linked to the onset of cognitive impairment and dementia are still poorly understood [26]. Relevant cortical areas for memorization and olfaction, such as temporal lobe regions (i.e., parahippocampal gyrus) and the middle and superior temporal gyri, have been reported to be altered in PD patients with cognitive decline [27,63]. Therewithal, it is also thought that at a certain level of olfactory loss, cognition may no longer play a crucial role in the interpretation of smell stimuli [27]. A study in PD patients with hyposmia revealed a dopaminergic denervation in the hippocampus and a reduction in cholinergic pathways in the archicortex, decreasing their ability to recognize odors [27,64].

More attention should be paid to the mechanisms involved in olfactory disorders, cognitive deficits, and possible biomarkers. Their analysis and understanding should lead to the implementation of specific protocols that could improve the early detection of various NDs, ensuring their better characterization and treatment, and, if this is the case, early diagnosis and intervention [59]. In addition to studies evaluated in Table 2 and Supplementary Table S5, further investigations with higher levels of evidence would help support the validation of hypothetical underlying mechanisms by providing contrasted experimental and clinical data.

### 4.7. Fundamental Bases of Olfactory Disorders through Neuroimaging

Neuroimaging tools are usually used to confirm the presence of NDs. Throughout this review, the correlation between structural brain changes and ODs has been analyzed. Magnetic resonance imaging (MRI) is the most common medical imaging test used to study the regions of interest in AD patients. MRI studies suggest that ODs can be good predictors of structural changes in the AD-signature cortex since it is significantly smaller in patients with ODs than in non-OD patients [38]. ODs were also associated with an atrophy of the medial temporal lobe, including the amygdala, hippocampal, and parahippocampal regions [25,38], thereby suggesting that ODs in MCI and AD patients are a consequence of impaired intracerebral olfactory processing [25].

In addition, MRI showed atrophic changes in the bilateral medial orbital cortex and the hippocampus, which correlated with OSIT-J scores in ALS, thus suggesting that the observed atrophy in the central olfactory system may explain ODs in ALS patients [42].

Moreover, diffusion tensor imaging (DTI) showed that PD patients with MCI and poorer olfactory outcomes exhibit more microstructural abnormalities than PD patients with no cognitive decline [36]. In addition, dopamine transporter single-photon emission computed tomography (DaT-SPECT) also explained that the high prevalence of olfactory loss in PD patients, as measured by the UPSIT, is due to the association of ODs with the pronounced loss of nigrostriatal dopamine neurons in the putamen and caudate nucleus [36]. Therewithal, more studies should investigate the association between atrophy degree in regions of interest in relation to ODs and other overlapping comorbidities.

### 4.8. Clinical Implications

The field of olfaction research suggests that olfactometries, in conjunction with other tests, could serve as specific diagnostic tools, potentially aiding in the confirmatory diagnosis of PD and AD [17]. Thus, the results of this review support the hypothesis that the use of olfactory tests may help not only in the early diagnosis but also in the characterization, monitorization, and rehabilitation of NDs. Olfactory training, although out of the scope of this review, has shown a decrease in depressive symptoms and an improvement in some cognitive functions, such as memory, attention, and speech, in patients with dementia [9].

Some clinical recommendations based on the information gathered in this review from our group are: (i) the use of standardized and validated smell tests to ensure the accuracy and comparability of the results; (ii) multimodal integration, combining olfactory assessment with other clinical variables, biomarkers, and neuropsychological tests for a comprehensive and accurate assessment of NDs; (iii) provide education and counseling to patients and family members on the importance and implications of olfactory loss in the context of dementia; and (iv) perform regular follow-ups to assess olfaction symptom progression and adjust the management plan as needed.

### 4.9. Limitations

The main limitation of our review is that due to the high heterogeneity of the studies included within a 6-year review period, a meta-analysis could not be performed, thereby restricting our findings to a qualitative analysis. Our results are also limited by the need for more qualified and accurate scientific studies; the lack of consensus on the optimal test to be used to assess olfaction in general and specifically in NDs; and the lack of homogeneity and comparability among studies. Moreover, not all the reviewed studies included a control group.

Additionally, some studies did not consider the presence of cofounding sinonasal disease factors, which may have biased the prevalence of olfactory alterations in NDs. Nevertheless, the prevalence of ODs was similar in studies that excluded patients with rhinological diseases (i.e., 86.1% for PD and 76.5% for AD/MCI) to those that did not exclude them (i.e., 82.8% for PD and 78.3% for AD). The reviewed meta-analyses did not report differences between studies associated with the presence of sinonasal diseases. Additionally, there is no proven relationship between nasal cavity diseases and the progression of cognitive impairment [2]. However, identifying treatable sinonasal conditions during the examination might improve the accuracy of olfactometry results in ND screening. Therefore, although we advocate for a multidisciplinary assessment when studying the role of olfaction in the early diagnosis of NDs (i.e., both rhinological and neuroscientific), current evidence at this point remains unclear.

## 5. Conclusions

Olfactory function is suggested to be an early or pre-clinical marker for neurodegenerative disease. Growing evidence suggests that deficits in smell function may constitute biomarkers for cognitive outcomes in patients with cognitive impairment and dementia. The conclusions derived from our systematic review, including 21 original articles and 6 meta-analyses focused on the predictive value of subjective olfactometry in the early detection and prognosis of neurodegenerative diseases such as Parkinson’s, Alzheimer’s, multiple sclerosis, and amyotrophic lateral sclerosis, are:

- The majority of studies reviewed found worse olfaction outcomes in patients with neurodegenerative diseases compared with healthy controls. They all agree to highlight a correlation between olfaction status and cognitive outcomes, thus suggesting olfactory impairment as a prognostic risk disease factor.
- Olfactory function and subdomains showed different peculiarities among different diseases. In Parkinson’s, the three domains of olfaction (identification, discrimination, and threshold) were affected, with various grades of severity being found among different Parkinson’s subtypes, whereas in Alzheimer’s, the most affected domain was odor discrimination. Changes in olfactory function, with peculiar characteristics among neurodegenerative diseases, may constitute useful markers for the better characterization and management of the disease.
- Subjective olfactometries that evaluate various olfaction domains, such as the Sniffin’ Sticks Test, are reported to provide more exhaustive information about olfactory function and its possible relationship with cognitive decline. In the future, the implementation and validation of new screening tests could help identify patient candidates for further olfactory evaluation, possibly facilitating its systematic adoption into ND routine assessments. Thus, a consensus on the methodology and optimal smell tests to be used in NDs must be reached.
- Future studies that analyze the predictive value and accuracy of subjective olfactometries and the specific alterations observed in different olfactory subdomains are still needed. Such studies should contribute with larger sample sizes and more data to increase and strengthen the current scientific evidence, thus possibly laying the basis for the definitive inclusion of olfactory protocols and testing in patients with dementia.

## Figures and Tables

**Table 1:** Follow-up, average sample size and age among the different groups of diseases reviewed.

Group Reviewed	Number of Studies	Average Sample Size	Largest Sample	Smaller Sample	Average Age (Weighted)
PD	13	155.7 ± 93.9	487	22	64.4 ± 3.0
AD	5	132.9 ± 149.9	265	52	70.2 ± 4.7
MS	2	56.3 ± 80.3	149	20	40.3 ± 2.0
ALS	1	30.0	-	-	67.3 ± 8.2

PD: Parkinson’s disease. AD: Alzheimer’s disease. MS: Multiple sclerosis. ALS: Amyotrophic lateral sclerosis.

## Data Availability

No new data were created or analyzed in this study. Data sharing is not applicable to this article.

## Supplementary Materials

The following supporting information can be downloaded at: https://www.mdpi.com/article/10.3390/life14030298/s1, Table S1: PRISMA check-list; Table S2: Summary of smell test used in the reviewed studies; Table S3: Cognitive batteries and tests used in the different studies reviewed; Table S4: Description of the cognitive domains assessed in each batterie or test and its main purpose; Table S5: Quality Assessment of case series studies from the National Institute for Health and Clinical Excellence (Appendix F) applied to this systematic review. References [43,51,52,54,55,56,65,66,67,68,69,70,71,72,73,74,75,76,77,78,79,80,81,82,83,84,85,86,87,88,89,90,91,92,93,94,95,96,97,98,99,100,101,102,103,104,105,106,107,108,109,110,111,112,113,114,115,116,117,118,119,120,121,122,123,124] are cited in the Supplementary Materials.
