# Supplementary material for: The Value of Subjective Olfactometry as a Predictive Biomarker of Neurodegenerative Diseases: A Systematic Review
Source: Life (Basel). 2024 Feb 23;14(3):298. doi: 10.3390/life14030298 (PMC10971131; doi:10.3390/life14030298)
Supplement: Supplementary file 1 [file life-14-00298-s001.zip › life-2850670-supplementary.pdf]

Supplementary Table S1. PRISMA check-list.

Section and Topic	Item	Checklist item	Location where item is reported
**TITLE**			
Title	1	Identify the report as a systematic review.	Page 1 (Lines 2-3)
**ABSTRACT**			
Abstract	2	See the PRISMA 2020 for Abstracts checklist.	Page 1 (Lines 13-28)
**INTRODUCTION**			
Rationale	3	Describe the rationale for the review in the context of existing knowledge.	Pages 1-2 (Lines 32-61)
Objectives	4	Provide an explicit statement of the objective(s) or question(s) the review addresses.	Page 2 (Lines 62-66)
**METHODS**			
Eligibility criteria	5	Specify the inclusion and exclusion criteria for the review and how studies were grouped for the syntheses.	Page 3 (Lines 102-111)
Information sources	6	Specify all databases, registers, websites, organizations, reference lists and other sources searched or consulted to identify studies. Specify the date when each source was last searched or consulted.	Page 2 (Lines 86-88)
Search strategy	7	Present the full search strategies for all databases, registers and websites, including any filters and limits used.	Pages 2-3 (Lines 75-85 and 88-100)
Selection process	8	Specify the methods used to decide whether a study met the inclusion criteria of the review, including how many reviewers screened each record and each report retrieved, whether they worked independently, and if applicable, details of automation tools used in the process.	Page 3 (Lines 113-116)
Data collection process	9	Specify the methods used to collect data from reports, including how many reviewers collected data from each report, whether they worked independently, any processes for obtaining or confirming data from study investigators, and if applicable, details of automation tools used in the process.	Page 3 (Lines 117-119)
Data items	10a	List and define all outcomes for which data were sought. Specify whether all results that were compatible with each outcome domain in each study were sought (e.g. for all measures, time points, analyses), and if not, the methods used to decide which results to collect.	Page 3 (Lines 117-119) and Tables S2, S3 and S4
	10b	List and define all other variables for which data were sought (e.g. participant and intervention characteristics, funding sources). Describe any assumptions made about any missing or unclear information.	Page 4 (Lines 117-119)
Study risk of bias assessment	11	Specify the methods used to assess risk of bias in the included studies, including details of the tool(s) used, how many reviewers assessed each study and whether they worked independently, and if applicable, details of automation tools used in the process.	Page 3 (Lines 121-127) and Table S5
Effect measures	12	Specify for each outcome the effect measure(s) (e.g. risk ratio, mean difference) used in the synthesis or presentation of results.	Page 4 (133-166) and Table S2, S3 and S4
Synthesis methods	13a	Describe the processes used to decide which studies were eligible for each synthesis (e.g. tabulating the study intervention characteristics and comparing against the planned groups for each synthesis (item #5)).	Page 3 (Lines 129-132)
	13b	Describe any methods required to prepare the data for presentation or synthesis, such as handling of missing summary statistics, or data conversions.	Page 3 (Lines 129-130) and Page 4 (133-165)
	13c	Describe any methods used to tabulate or visually display results of individual studies and syntheses.	Pages 3-4 (Lines 129-137)
	13d	Describe any methods used to synthesize results and provide a rationale for the choice(s). If meta-analysis was performed, describe the model(s), method(s) to identify the presence and extent of statistical heterogeneity, and software package(s) used.	Pages 3-4 (Lines 129-137)
	13e	Describe any methods used to explore possible causes of heterogeneity among study results (e.g. subgroup analysis, meta-regression).	Not applicable
	13f	Describe any sensitivity analyses conducted to assess robustness of the synthesized results.	Not applicable
Reporting bias assessment	14	Describe any methods used to assess risk of bias due to missing results in a synthesis (arising from reporting biases).	Page 3 (Lines 121-127)
Certainty assessment	15	Describe any methods used to assess certainty (or confidence) in the body of evidence for an outcome.	Table S5

Supplementary Table S1. PRISMA check-list.

Section and Topic	Item	Checklist item	Location where item is reported
**RESULTS**			
Study selection	16a	Describe the results of the search and selection process, from the number of records identified in the search to the number of studies included in the review, ideally using a flow diagram.	Pages 4-5 (Lines 168-173) and Figure 1
	16b	Cite studies that might appear to meet the inclusion criteria, but which were excluded, and explain why they were excluded.	Figure 1
Study characteristics	17	Cite each included study and present its characteristics.	Tables 1, 2 and 3
Risk of bias in studies	18	Present assessments of risk of bias for each included study.	Table S5
Results of individual studies	19	For all outcomes, present, for each study: (a) summary statistics for each group (where appropriate) and (b) an effect estimate and its precision (e.g. confidence/credible interval), ideally using structured tables or plots.	Tables 2 and 3
Results of syntheses	20a	For each synthesis, briefly summarize the characteristics and risk of bias among contributing studies.	Pages 6-7 (Lines 196-273) and Table S5
	20b	Present results of all statistical syntheses conducted. If meta-analysis was done, present for each the summary estimate and its precision (e.g. confidence/credible interval) and measures of statistical heterogeneity. If comparing groups, describe the direction of the effect.	Not applicable
	20c	Present results of all investigations of possible causes of heterogeneity among study results.	Tables 2 and 3
	20d	Present results of all sensitivity analyses conducted to assess the robustness of the synthesized results.	Not applicable
Reporting biases	21	Present assessments of risk of bias due to missing results (arising from reporting biases) for each synthesis assessed.	Not applicable
Certainty of evidence	22	Present assessments of certainty (or confidence) in the body of evidence for each outcome assessed.	Tables 2 and 3
**DISCUSSION**			
Discussion	23a	Provide a general interpretation of the results in the context of other evidence.	Pages 13-17 (Lines 278-479)
	23b	Discuss any limitations of the evidence included in the review.	Page 17 (Lines 497-517)
	23c	Discuss any limitations of the review processes used.	Page 17 (Lines 497-503)
	23d	Discuss implications of the results for practice, policy, and future research.	Page 17 (Lines 481-495)
**OTHER INFORMATION**			
Registration and protocol	24a	Provide registration information for the review, including register name and registration number, or state that the review was not registered.	Not registered
	24b	Indicate where the review protocol can be accessed, or state that a protocol was not prepared.	Page 2 (Line 70)
	24c	Describe and explain any amendments to information provided at registration or in the protocol.	Not applicable
Support	25	Describe sources of financial or non-financial support for the review, and the role of the funders or sponsors in the review.	Page 18 (Line 566-569)
Competing interests	26	Declare any competing interests of review authors.	Page 19 (Line 585)
Availability of data, code and other materials	27	Report which of the following are publicly available and where they can be found: template data collection forms; data extracted from included studies; data used for all analyses; analytic code; any other materials used in the review.	Page 18 (Lines 577-578)

Supplementary Table S2. Summary of smell test used in the reviewed studies.

Author	Year of publication	Test name / Country of validation	Smell domains analyzed	Confounding factors analyzed	Method of execution
Doty et al. [43]	1984<br>1985	University of Pennsylvania Smell Identification Test (UPSIT)<br><br>EE.UU.	Identification	Age, sex, culture, smoking, sinonasal disease, cognitive impairments	Number of odorants: 40<br>Choice: A smell must be chosen from a list of 4 options<br>Type of test: Olfactometry of printed odorants<br>Completion time: 15 minutes<br>Peculiarities: It is the test with the highest test-retest reliability reported (r=0.94)
Toyota et al. [65,66]	1978<br>2004*	T&T Olfactometer<br><br>Jet Stream Olfactometer (JSO)*<br><br>Japan	Threshold<br>Identification	Age, sex, sinonasal disease	Number of odorants: 5<br>Choice: 5 odorants at different concentrations must be named<br>Type of test: Odorless filter paper impregnated with liquid odorants<br>Completion time: Not defined<br>Peculiarities: It analyzes threshold and identification, with the same 5 odorants<br>*JSO is a newly developed smell stimulus device, designed to reduce possible odor contamination
Murphy et al. [67]	1994	San Diego Odour Identification Test (S-DOIT)<br><br>EE.UU.	Identification	Age	Number of odorants: 6<br>Choice: A smell must be chosen from a list of 20 options through visual stimuli: 6 right choices and 14 distractors<br>Type of test: Olfactometry of liquid odorants in bottles<br>Completion time: NR<br>Peculiarities: Easy, inexpensive, it avoids linguistic difficulties and does not require reading skills
Doty et al. [51]	1995<br>1996	Cross – Cultural Smell Identification Test (CC–SIT)<br><br>Brief Smell Identification Test (B-SIT)<br><br>EE.UU., Europe, Asia	Identification	Age, sex	Number of odorants: 12<br>Choice: A smell must be chosen from a list of 4 options<br>Type of test: Olfactometry of printed odorants<br>Completion time: 5 minutes<br>Peculiarities: It is frequently used as a screening tool to select patients candidates for UPSIT test
Hummel et al.<br>***Kobal et al.*** [54]	1997<br>2000	Sniffin' Sticks Test (SST)<br><br>Germany, Switzerland, Austria, Australia, Italy, EE.UU.	Threshold<br>Discrimination<br>Identification	Age	Number of odorants: 33 (1 for threshold, 16 for discrimination and 16 for identification)<br>Choice: A smell must be chosen from a list of options (3 options for threshold and discrimination; 4 options for identification)<br>Type of test: Liquid odorant pens<br>Completion time: Not defined<br>Peculiarities: It is the only test that analyzes three domains of smell: 1) Threshold by simple staircase method for detection; 2) Discrimination: 16 odorants in trios for different odor detection; 3) Identification: 16 odorants among a list of 4 options.
Parola and Liberini [68]	1999	Culturally adapted smell identification test (CA-SIT)<br><br>Italy	Identification	Age, sex, culture, smoking, sinonasal disease, cognitive impairments	Number of odorants: 34<br>Choice: Named, yes-no and identification of a smell from a list of 4 options<br>Type of test: Olfactometry of printed odorants<br>Completion time: Not defined<br>Peculiarities: Three steps: 1) Odor naming test: provide a name for each of a set of odorants; 2) Yes-no odor identification test: determine whether a stimulus smells like a particular odorant (i.e. does this smell like a rose?); 3) Multiple choice odor identification test: identify the stimulus from a list odorants

Hummel et al. [55]	2001	Sniffin Sticks test 12 items (SST-12)<br><br>Germany	Identification	Age, sex	Number of odorants: 12<br>Choice: A smell must be chosen from a list of 4 options<br>Type of test: Liquid odorant pens<br>Completion time: 4 minutes<br>Peculiarities: It is frequently used as a screening tool to select patients candidates for the SST test
Saito et al. [69]	2006	Odor Stick Identification Test for the Japanese (OSIT-J)	Identification<br>Discrimination	Age, sex	Number of odorants: 13<br>Choice: A smell must be chosen from a list of 4 options<br>Type of test: Liquid odorant pens<br>Completion time: 4 minutes<br>Peculiarities: Validated for Japanese population
Hoo Cho et al. [70]	2009	Korean Sniffin' Sticks Test (KVSS)<br><br>Korea	Threshold<br>Discrimination<br>Identification	Age	Number of odorants: 33 (1 for threshold, 16 for discrimination and 16 for identification)<br>Choice: A smell must be chosen from a list of 3 options for threshold and discrimination; 4 options for identification<br>Type of test: Liquid odorant pens<br>Completion time: Not defined<br>Peculiarities: Similar to SST but with 4 modified odorants (vanilla, resin, soy and sesame oil) adapted to Korean population
Okutani et al. [52]	2013	Open Essence (OE)<br><br>Japan	Identification	Age, sex	Number of odorants: 12<br>Choice: A smell must be chosen from a list of 6 options<br>Type of test: Olfactometry of printed odorants<br>Completion time: Not defined<br>Peculiarities: It is useful as a screening test before T&T olfactometry
Taherkhani et al. [71]	2014	Iran Smell Identification Test (Iran-SIT)	Identification	Age, sex, culture, smoking, sinonasal disease, cognitive impairments	Number of odorants: 24<br>Choice: A smell must be chosen from a list of 4 options<br>Type of test: Olfactometry of printed odorants<br>Completion time: 12 minutes<br>Peculiarities: Validated only for Iranian population
Lawton et al. [56]	2016	Sniffin Sticks test 16 items (SST-16)<br><br>United Kingdom	Identification	Age, sex, cognitive impairments	Number of odorants: 12<br>Choice: A smell must be chosen from a list of 4 options<br>Type of test: Liquid odorant pens above a paraffin paper<br>Completion time: 4 minutes<br>Peculiarities: It is used as a screening tool to select patients candidates for the SST. Specifically validated for NDs
Dhilla-Albers et al. [72]	2016	Odor Percept Identification (OPID)<br><br>EE.UU.	Identification	Age, sex, educational level, sinonasal disease, previous nasal intervention	Number of odorants: 30<br>Choice: A smell must be chosen from a list of 4 options<br>Type of test: Smell stimulus device<br>Completion time: Not defined<br>Peculiarities: Two steps: 1) OPID-10: 10 odors selected for their predictive ability to detect progression from MCI to AD. 2) OPID-20: OPID-10 + 10 novel odors.

Supplementary Table S3. Cognitive batteries and tests used in the different studies reviewed.

Author and year of publication	Neurodegenerative disease	Batteries and tests	Scoring
Camargo et al., 2018	PD	SCOPA-Cog<br>MMSE	≥ 22/43 = absence of dementia.<br>≥ 26/30 = absence of dementia.
Masala et al., 2018	PD	MoCA	≥ 26/30 = normal cognition
Yoo et al., 2019	PD	SNSB<br>K-MMSE<br>CDR-SOB	Impairment in: < than two items = intact cognition; ≥ two items, subjective cognitive complaint, no abnormal ADL = MCI; two items and fulfilled the clinical criteria = PDD
Roos et al., 2019	PD	MMSE	Lower the score more severe cognitive loss
Yoshii et al.,2019	AD<br>MCI	ADAS-Jcog	The maximum score is 70. A higher score indicates poor performance
Lian et al.,2019	AD	MMSE<br>AVLT<br>BNT<br>RCFT<br>SDMT<br>SCWTC	Higher scores indicate better cognitive performance, except for SCWT.
Doorduijn et al., 2020	AD<br>MCI	VAT<br>RAVLT<br>TMT A<br>Digit span<br>SCWT<br>Word fluency (letter & category)<br>Dot counting VOSP<br>Fragmented letters VOSP<br>Number location VOSP<br>MMSE	Higher scores indicate better cognitive performance, except for TMT A, and SCWT.
Da Silva et al., 2020	MS	MMSE	Lower scores indicates poorer cognition ability.
Fujio et al., 2020	PD	K-MMSE	Maximum score being 30 points, ≥ 28 = normal, ≤ 27 = suspected MCI, and ≤ 23 = suspected dementia.
Lee et al., 2021	PD	K-MoCA<br>K-MMSE	N/A
Masuda et al., 2021	ALS	RCPM<br>3MS<br>FAB<br>SCWT<br>Digit Span WAIS-III<br>Word fluency (letter & category)<br>Recognition ADAS-Jcog<br>Picture naming SALA<br>Noun similarity judgment SALA	N/A
Duz et al., 2021	RRMS<br>RIS	SMMT<br>VMLT<br>Visual reproduction WMS<br>Digit Span WMS<br>Letter, category, and alternative fluency WMS & SWCT<br>BJLO<br>BFRT	N/A
Wang et al., 2021	AD<br>MCI	MMSE<br>MES<br>AVLT<br>BNT<br>RCFT<br>SMDT<br>TMT	N/A
Trentin et al., 2022	PD	MoCA	N/A
Saunders-Pullman et al., 2022	PD	MoCA<br>UPDRS I	N/A
Thomas et al., 2022	MCI<br>LBD<br>AD	ACE-R<br>MMSE	
Almeida et al., 2022	PD	MMSE	Exclusion criteria:<br>≤ 20 and illiterate or ≤ 24 with more than four years or an unknown year of education.

Nabizadeh et al., 2022	PD	MoCA<br>SDMT<br>Judgment of Line Orientation<br>LNS<br>Semantic fluency	N/A
Stewart et al., 2023	PD	Block Design WASI<br>CVLT<br>SDMT<br>Verbal Fluency D-KEFS<br>TMT D-KEFS<br>TMT<br>Spatial Span WMS-III	Impairment on ≥ 2 tests = PD MCI

ACE-R: Addenbrooke's Cognitive Examination Revised; AD: Alzheimer's Disease; ADAS-Jcog: AD Assessment Scale-Cognitive Subscale, Japanese Version; ADL: activities of daily living; ALS: Amyotrophic lateral sclerosis; AVLT: Auditory Verbal Learning Test; BFRT: Benton Facial Recognition Test; BJLOT: Benton Judgment of Line Orientation Test; BNT: Boston Naming Test; ; CDR-SOB: Clinical Dementia Rating - Sum of Boxes; CVLT: The California Verbal Learning Test; D-KEFS: Delis Kaplan Executive Function System; FAB: frontal assessment battery; K-MoCA: Korean version of the Montreal Cognitive Assessment; K-MMSE: Korean version of the Mini-Mental State Examination; LNS: Letter Number Sequencing; LBD: Lewy Bodies Disease; MCI: Mild Cognitive Impairment; MES: Memory and Executive Screening; MMSE: Mini-Mental State Examination; MoCA: Montreal Cognitive Assessment; MS: Multiple Sclerosis; PD: Parkinson's Disease; PDD: Parkinson's disease dementia; RAVLT: Rey auditory verbal learning task; RCFT: Rey-Osterrieth Complex Figure Test; RCPM: Raven's Colored Progressive Matrices; RIS: Radiologic isolated syndrome; RRMS: Relapsing remitting multiple sclerosis; SCOPA-Cog: Scales for Outcomes in Parkinson's Disease-Cognition; SCWT: Stroop Color-Word Test; SALA: Sophia Analysis of Language in Aphasia; SCWT: Stroop Color-Word Test-Chinese version; SDMT: Symbol Digit Modality Test; SMMT: Standardized mini-mental test; SNSB: Seoul Neuropsychological Screening Battery; TMT: Trail Making Test; UPDRS: Unified Parkinson Disease Rating Scale; VAT: Visual association test; VMLT: verbal memory learning test; WAIS-III: Wechsler Adult Intelligence Scale-III; WASI: Wechsler Abbreviated Scale of Intelligence; WMS: Wechsler Memory Scale; 3MS: Modified Mini-Mental State examination

Supplementary Table S4. Description of the cognitive domains assessed in each batterie or test and its main purpose.

Name of the test	Cognitive Domains Assessed	Purpose	References
**ACE-R**	Orientation/attention, memory, verbal fluency, language, and visuospatial ability	Brief cognitive screening assessment that is sensitive to the early stages of dementia and able to differentiate between dementia subtypes.	[73]
**ADAS-Jcog**	Memory, orientation, language, and praxis	Japanese version of ADAS-Cog to assess the level of cognitive dysfunction in Alzheimer's disease.	[74,75]
**AVLT**	Immediate, delayed, and total verbal memory	Frequently used in neuropsychology literature to comprehensively assess the memory and discriminate MCI.	[76]
**BFRT**	Face discrimination and recognition abilities	Identify individuals with prosopagnosia.	[77,78]
**BJLOT**	Visuospatial judgment	Detects right hemisphere dysfunction. It's application just needs verbal response, avoiding contamination from constructional and motor speed factors.	[79,80]
**BNT**	Naming and lexical retrieval	Identify lexical retrieval difficulties.	[81-83]
**Block design**	Spatial visualization ability and motor skill.	Assessment of human intelligence.	[84]
**CDR-SOB**	Memory, orientation, judgment, community affairs, home hobbies, and personal care	Diagnostic tool for staging dementia due to AD.	[85-86]
**CVLT**	Verbal inhibition, retention, encoding, and retrieval abilities.	Measuring cognitive changes and disease progression.	[87-88]
**D-KEFS**	Verbal and nonverbal executive functions	Standardized assessment of higher-level cognitive functions, which evaluates mild brain damage in the frontal lobe.	[89]
**Digit Span**	Short term verbal memory, and working memory	Measures overall intelligence.	[90,91]
**FAB**	Conceptualization, mental flexibility, and motor programming	Identify patients with frontal lobe lesions, assessing the presence and severity of a dysexecutive syndrome affecting both cognition and motor behavior.	[92]
**K-MoCA**	Visuospatial and executive function, naming, memory, attention, language, abstraction, and orientation	Korean version of the Montreal Cognitive Assessment.	[93,94]
**K-MMSE**	Time orientation, spatial orientation, memory registration, attention and calculation, memory recall, language, and space-time configuration	Korean version of the Mini-Mental State Examination.	[95]
**LNS**	Working memory capacity	Capacity to temporarily store and manipulate information.	[90,96]
**MES**	Memory and executive function	Global screening method for MCI and mild dementia.	[97]
**MMSE**	Orientation, registration, attention/calculation, recall, and language	Global screening instrument for dementia.	[98,99]
**MoCA**	Visuospatial and executive function, naming, memory, attention, language, abstraction, and orientation	Global screening tool that provides a chance to detect subtle cognitive impairment at early stages.	[93]
**RAVLT**	Verbal inhibition, retention, encoding, and retrieval abilities	Detect changes associated with abnormal aging.	[100]
**RCFT**	Visuospatial ability, visual memory, and executive functions	Useful tool for the evaluation of neuropsychological functions and brain dysfunction in charge of the occipital–parietal lobe and the prefrontal lobe.	[101,102]
**RCPM**	Abstract reasoning and thinking	Non-verbal intelligence test.	[103,104]
**SCOPA-Cog**	Memory, attention, executive function, and visuospatial function.	Short, reliable, and valid instrument for assessing cognitive function in PD.	[105]
**SALA**	Language	Japanese assessment to analyze language impairment in patients with aphasia.	[106]

**SCWT**	Inhibition, attention, processing speed, cognitive flexibility and working memory.	Studies populations with brain damage and mental disorders such as dementia, depression, and ADHD.	[107,108]
**SDMT**	Divided attention, perceptual speed, visual scanning speed, and tracking	Detect cognitive change associated with numerous neurological conditions and acquired brain injury.	[109,110]
**SMMT**	Orientation to time and place, registration, concentration, short-term recall, naming familiar items, repeating a common expression, and the ability to read and follow written instructions, write a sentence, construct a diagram, and follow a three-step verbal command.	Comprehensive assessments of older adults, pinpointing specific deficits that can aid in forming a diagnosis.	[111]
**SNSB**	Attention, language, visuospatial, memory, and frontal executive function	Neuropsychological tests in Korea for assessing cognitive functions in patients with stroke, head trauma, PD, and dementia.	[112]
**Spatial span**	Visuospatial working memory	Assess non-verbal memory deficits in patients with damage to the parieto-occipital lobes of the brain.	[90]
**TMT**	Executive function, psychomotor speed, and visual scanning	Sensitive test for the presence of brain injury.	[113,114]
**UPDRS I**	Mentation, behavior, and mood	Follow the longitudinal course of PD.	[115]
**VAT**	Learning	Discriminate between early dementia of the Alzheimer type, other type of dementia, and non-demented people.	[116,117]
**Visual reproduction**	Immediate and delayed visual reproduction	Detect and evaluate memory disorders.	[118,119]
**VMLT**	Verbal memory and learning abilities	Measures cognitive decline in the early phases of neurocognitive impairment.	[120]
**VOSP**	Visuospatial functions	To distinguish the clinical symptoms of various disease types, but mainly neurocognitive disorders whose pathology involves the visuoperceptual function.	[121]
**Word fluency (letter, category & alternative)**	Verbal ability and executive control to verbal fluency.	Short test of verbal functioning used to support diagnoses of ADHD and cognitive impairment.	[122,123]
**3MS**	Attention, concentration, orientation to time and place, long-term and short-term memory, language ability, constructional praxis, abstract thinking, and list-generating fluency.	Screening test for cognitive loss or a brief bedside cognitive assessment.	[124]

ACE-R: Addenbrooke's Cognitive Examination Revised; AD: Alzheimer's Disease; ADAS-Jcog: AD Assessment Scale-Cognitive Subscale, Japanese Version; ADHD: attention-deficit/hyperactivity disorder; AVLT: Auditory Verbal Learning Test; Benton BFRT:Facial Recognition Test; BJLOT: Benton Judgment of Line Orientation Test; BNT: Boston Naming Test; CDR-SOB: Clinical Dementia Rating - Sum of Boxes; CVLT: The California Verbal Learning Test; D-KEFS: Delis Kaplan Executive Function System; FAB: Frontal assessment battery; K-MoCA: Korean version of the Montreal Cognitive Assessment; K-MMSE: Korean version of the Mini-Mental State Examination; LNS: Letter Number Sequencing; MCI: Mild Cognitive Impairment; MES: Memory and Executive Screening; MMSE: Mini-Mental State Examination; MoCA: Montreal Cognitive Assessment; PD: Parkinson's Disease; RAVLT: Rey auditory verbal learning task; RCFT: Rey-Osterrieth Complex Figure Test; RCPM; Raven's Colored Progressive Matrices; SCOPA-Cog: Scales for Outcomes in Parkinson's Disease-Cognition; SCWT: Stroop Color-Word Test; SALA: Sophia Analysis of Language in Aphasia; SDMT: Symbol Digit Modality Test; SMMT: Standardized mini-mental test; SNSB: Seoul Neuropsychological Screening Battery; TMT: Trail Making Test; UPDRS: Unified Parkinson Disease Rating Scale; VAT: Visual association test; VMLT: verbal memory learning test; 3MS: Modified Mini-Mental State examination

Supplementary Table S5. Quality Assessment of case series studies from the National Institute for Health and Clinical Excellence (Appendix F) applied to this systematic review.

	1.1	1.2	1.3	2.1	2.2	2.3	2.4	2.5	2.6	2.7	2.8	2.9	2.10	3.1	3.2	3.3	3.4	3.5	3.6	4.1	4.2	4.3	4.4	4.5	4.6	5.1	5.2
**Camargo *et al*, 2018**	+	-	+	+	++	NA	NA	NA	NA	NA	NA	NA	NA	+	+	+	+	NA	NA	NA	NA	NR	++	+	++	+	-
**Masala *et al*, 2018**	+	+	+	+	++	NA	NA	NA	NA	NA	NA	NA	NA	+	+	+	+	NA	NA	NA	NA	NR	++	+	++	+	-
**Jalali *et al*, 2019**	+	+	+	+	+	NA	NA	NA	NA	NA	NA	NA	NA	+	+	-	+	NA	NA	NA	NA	++	+	+	+	+	-
**Yoo *et al*, 2019**	++	++	-	+	++	NA	NA	NA	NA	NA	NA	NA	NA	++	++	++	+	++	++	++	NA	NR	++	++	+	+	-
**Roos *et al*, 2019**	+	+	-	-	++	NA	NA	NA	NA	NA	NA	NA	NA	+	++	+	+	NA	NA	NA	NA	NR	+	+	++	-	-
**Yoshii *et al*, 2019**	+	+	-	+	+	NA	NA	NA	NA	NA	NA	NA	NA	-	+	+	+	NA	NA	NA	NA	NR	++	+	+	-	-
**Lian *et al*, 2019**	+	-	+	+	++	NA	NA	NA	NA	NA	NA	NA	NA	+	++	++	+	NA	NA	NA	NA	NR	++	++	++	-	-
**Doorduijn *et al*, 2020**	+	-	+	+	++	NA	NA	NA	NA	NA	NA	NA	NA	+	++	+	+	NA	NA	NA	NA	NR	++	+	++	-	-
**Da Silva *et al*, 2020**	++	+	+	+	+	NA	NA	NA	NA	NA	NA	NA	NA	++	+	+	+	NA	+	NA	NA	NR	++	++	+	+	-
**Fujio *et al*, 2020**	+	+	-	+	++	NA	NA	NA	NA	NA	-	NA	NA	-	+	-	+	NA	-	NA	+	NR	+	+	+	-	-
**Lee *et al*, 2021**	+	-	+	+	++	NA	NA	NA	NA	NA	NA	NA	NA	+	+	+	+	NA	-	NA	NA	NR	++	+	++	+	-
**Masuda *et al*, 2021**	+	+	-	+	+	NA	NA	NA	NA	NA	NA	NA	NA	+	++	++	+	NA	NA	NA	NA	NR	+	+	+	-	-
**Duz *et al*, 2021**	+	-	+	+	++	NA	NA	NA	NA	NA	NA	NA	NA	+	+	+	+	NA	NA	NA	NA	NR	+	+	++	-	-
**Elhassanien *et al*, 2021**	-	-	-	+	++	NA	NA	NA	NA	NA	NA	NA	NA	+	-	-	+	NA	NA	NA	NA	NR	++	+	++	-	-
**Wang *et al*, 2021**	+	+	-	+	++	NA	NA	NA	NA	NA	NA	NA	NA	+	+	++	+	NA	NA	NA	NA	NR	++	++	++	+	-
**Trentin *et al*, 2022**	++	++	+	+	++	NA	NA	NA	NA	NA	NA	NA	NA	+	+	++	+	NA	NA	NA	NA	NR	+	++	++	-	-
**Saunders-Pullman *et al*, 2022**	+	+	-	-	+	NA	NA	NA	NA	NA	-	NA	NA	+	+	++	+	+	-	+	+	NR	+	+	+	++	-
**Thomas *et al*, 2022**	-	-	-	+	+	NA	NA	NA	NA	NA	NA	NA	NA	-	+	+	+	NA	NA	NA	NA	NR	+	++	+	+	-
**Almeida *et al*, 2022**	+	+	+	+	+	NA	NA	NA	NA	NA	NA	NA	NA	++	+	++	+	NA	NA	NA	NA	NR	+	+	+	-	-
**Nabizadeh *et al*, 2022**	++	+	-	+	++	NA	NA	NA	NA	NA	NR	NA	NA	+	+	++	+	+	+	++	NR	NR	+	++	++	+	-
**Stewart *et al*, 2023**	+	+	-	-	++	NA	NA	NA	NA	NA	NA	NA	NA	-	+	++	+	NA	NA	NA	NA	NR	+	++	++	-	-

Questions of Appendix F ✢ Section 1: Population. 1.1 Is the source population or source area well described? **1.2** Is the eligible population or area representative of the source population or area? **1.3** Do the selected participants or areas represent the eligible population or area? **Section 2: Method of allocation to intervention (or comparison). 2.1** Allocation to intervention (or comparison). How was selection bias minimized? **2.2** Were interventions (and comparisons) well described and appropriate? **2.3** Was the allocation concealed? **2.4** Were participants or investigators blind to exposure and comparison? **2.5** Was the exposure to the intervention and comparison adequate? **2.6** Was contamination acceptably low? **2.7** Were other interventions similar in both groups? **2.8** Were all participants accounted for at study conclusion? **2.9** Did the setting reflect usual UK practice? **2.10** Did the intervention or control comparison reflect usual UK practice? **Section 3: Outcomes. 3.1** Were outcome measures reliable? **3.2** Were all outcome measurements complete? **3.3** Were all important outcomes assessed? **3.4** Were outcomes relevant? **3.5** Were there similar follow-up times in exposure and comparison groups? **3.6** Was follow-up time meaningful? **Section 4: Analyses. 4.1** Were exposure and comparison groups similar at baseline? If not, were these adjusted? **4.2** Was intention to treat (ITT) analysis conducted? **4.3** Was the study sufficiently powered to detect an intervention effect (if one exists)? **4.4** Were the estimates of effect size given or calculable? **4.5** Were the analytical methods appropriate? **4.6** Was the precision of intervention effects given or calculable? Were they meaningful? **Section 5: Summary. 5.1** Are the study results internally valid (i.e. unbiased)? **5.2** Are the findings generalizable to the source population (i.e. externally valid)?

++ Indicates that for that particular aspect of study design, the study has been designed or conducted in such a way as to minimize the risk of bias. **+** Indicates that either the answer to the checklist question is not clear from the way the study is reported, or that the study may not have addressed all potential sources of bias for that particular aspect of study design. **–** Should be reserved for those aspects of the study design in which significant sources of bias may persist. **NR (not reported)** Should be reserved for those aspects in which the study under review fails to report how they have (or might have) been considered. **NA (not applicable)** Should be reserved for those study design aspects that are not applicable given the study design under review (for example, allocation concealment would not be applicable for case control studies).

References

(43) Doty, R. L.; Shaman, P.; Kimmelman, C. P.; Dann, M. S. University of Pennsylvania Smell Identification Test: A Rapid Quantitative Olfactory Function Test for the Clinic. *The Laryngoscope* **1984**, *94* (2), 176–178.

(51) Doty, R. L.; Marcus, A.; William Lee, W. Development of the 12-Item Cross-Cultural Smell Identification Test (CC-SIT). *The Laryngoscope* **1996**, *106* (3), 353–356.

(52) Okutani, F.; Hirose, K.; Kobayashi, T.; Kaba, H.; Hyodo, M. Evaluation of "Open Essence" Odor-Identification Test Card by Application to Healthy Volunteers. *Auris Nasus Larynx* **2013**, *40* (1), 76–80.

(54) Kobal, G.; Hummel, T.; Sekinger, B.; Barz, S.; Roscher, S.; Wolf, S. "Sniffin'sticks": Screening of Olfactory Performance. *Rhinology* **1996**, *34* (4), 222–226.

(55) Hummel, T.; Rosenheim, K.; Konnerth, C.-G.; Kobal, G. Screening of Olfactory Function with a Four-Minute Odor Identification Test: Reliability, Normative Data, and Investigations in Patients with Olfactory Loss. *Annals of Otology, Rhinology & Laryngology* **2001**, *110* (10), 976–981.

(56) Lawton, M.; Hu, M. T.; Baig, F.; Ruffmann, C.; Barron, E.; Swallow, D. M.; Malek, N.; Grosset, K. A.; Bajaj, N.; Barker, R. A.; others. Equating Scores of the University of Pennsylvania Smell Identification Test and Sniffin'Sticks Test in Patients with Parkinson's Disease. *Parkinsonism & related disorders* **2016**, *33*, 96–101.

(65) Takagi, S.; Toyota, B.; Kitamura, T. Olfactory Disorders: Measurements and Treatments. *Tokyo Igakushoin* **1978**, *8*.

(66) Miwa, T.; Furukawa, M.; Matsune, S.; Kurono, Y.; Harada, H.; Kato, T.; Tokumaru, H.; Nakashima, T.; Ono, N.; Yamashita, H.; others. Clinical Usefulness of Five Odorant Jet Stream Olfactometry. *Nihon Bika Gakkai Kaishi (Japanese Journal of Rhinology)* **2004**, *43* (2), 182–187.

(67) Murphy, C.; Anderson, J. A.; Markison, S. Psychophysical Assessment of Chemosensory Disorders in Clinical Populations. In *Olfaction and Taste XI: Proceedings of the 11th International Symposium on Olfaction and Taste and of the 27th Japanese Symposium on Taste and Smell. Joint Meeting held at Kosei-nenkin Kaikan, Sapporo, Japan, July 12–16, 1993*; Springer, 1994; pp 609–613.

(68) Parola, S.; Liberini, P. Assessing Olfaction in the Italian Population: Methodology and Clinical Application. *The Italian Journal of Neurological Sciences* **1999**, *20*, 287–296.

(69) Saito, S. Development of an Odor Identification Test for Japanese People: Verification of Stick Type and Card Type. *J Jpn Assoc Odor Environ* **2003**, *34*, 1.

(70) Cho, J. H.; Jeong, Y. S.; Lee, Y. J.; Hong, S.-C.; Yoon, J.-H.; Kim, J. K. The Korean Version of the Sniffin'stick (KVSS) Test and Its Validity in Comparison with the Cross-Cultural Smell Identification Test (CC-SIT). *Auris Nasus Larynx* **2009**, *36* (3), 280–286.

(71) Taherkhani, S.; Moztarzadeh, F.; Mehdizadeh Seraj, J.; Hashemi Nazari, S. S.; Taherkhani, F.; Gharehdaghi, J.; Okazi, A.; Pouraghaei, S. Iran Smell Identification Test (Iran-SIT): A Modified Version of the University of Pennsylvania Smell Identification Test (UPSIT) for Iranian Population. *Chemosensory perception* **2015**, *8*, 183–191.

(72) Dhilla Albers, A.; Asafu-Adjei, J.; Delaney, M. K.; Kelly, K. E.; Gomez-Isla, T.; Blacker, D.; Johnson, K. A.; Sperling, R. A.; Hyman, B. T.; Betensky, R. A.; others. Episodic Memory of Odors Stratifies Alzheimer Biomarkers in Normal Elderly. *Annals of neurology* **2016**, *80* (6), 846–857.

(73) Mioshi, E.; Dawson, K.; Mitchell, J.; Arnold, R.; Hodges, J. R. The Addenbrooke's Cognitive Examination Revised (ACE-R): A Brief Cognitive Test Battery for Dementia Screening. *International Journal of Geriatric Psychiatry* **2006**, *21* (11), 1078–1085. https://doi.org/10.1002/gps.1610.

(74) Honma, A. Development of a Japanese Version of Alzheimer's Disease Assessment Scale (ADAS). *Jpn J Geriatr Psychiatry* **1992**, *3*, 647–655.

(75) Kueper, J. K.; Speechley, M.; Montero-Odasso, M. The Alzheimer's Disease Assessment Scale–Cognitive Subscale (ADAS-Cog): Modifications and Responsiveness in Pre-Dementia Populations. a Narrative Review. *Journal of Alzheimer's Disease* **2018**, *63* (2), 423–444.

(76) Guo, Q.; Zhao, Q.; Chen, M.; Ding, D.; Hong, Z. A Comparison Study of Mild Cognitive Impairment with 3 Memory Tests among Chinese Individuals. *Alzheimer Dis Assoc Disord* **2009**, *23* (3), 253–259. https://doi.org/10.1097/WAD.0b013e3181999e92.

(77) Benton, A. L., Sivan, A. B., Hamsher, K. D. S., Varney, N. R., & Spreen, O. (**1983**). Facial recognition: Stimulus and multiple-choice pictures. In A. L. Benton, A. B. Sivan, K. D. S. Hamsher, N. R. Varney, & O. Spreen (Eds.), Contribution to neuropsychological assessment (pp. 30–40). Oxford University Press.enton, A.; Sivan, A.; Hamsher, K.; Varney, N.; Spreen, O. Benton Facial Recognition Test.

(78) Murray, E.; Bennetts, R.; Tree, J.; Bate, S. An Update of the Benton Facial Recognition Test. *Behav Res* **2022**, *54* (5), 2318–2333. https://doi.org/10.3758/s13428-021-01727-x.

(79) Benton, A. L.; Varney, N.; Hamsher, K. deS. Judgment of Line Orientation. **1983**.

(80) Hudon, C.; Belleville, S.; Belzile, F.; Landry, M.; Mulet-Perreault, H.; Trudel, C.; Macoir, J. Normative Data for the Judgment of Line Orientation Test (Long and Short Forms) in the Quebec-French Population Aged between 50 and 89 Years. *Archives of Clinical Neuropsychology* **2023**, *acad077*. https://doi.org/10.1093/arclin/acad077.

(81) Cheung, R.; Cheung, M.-C.; Chan, A. Confrontation Naming in Chinese Patients with Left, Right or Bilateral Brain Damage. *Journal of the International Neuropsychological Society : JINS* **2004**, *10*, 46–53. https://doi.org/10.1017/S1355617704101069.

(82) Jefferson, A. L.; Wong, S.; Gracer, T. S.; Ozonoff, A.; Green, R. C.; Stern, R. A. Geriatric Performance on an Abbreviated Version of the Boston Naming Test. *Appl Neuropsychol* **2007**, *14* (3), 215–223. https://doi.org/10.1080/09084280701509166.

(83) Li, E. C.; Williams, S. E. An Investigation of Naming Errors Following Semantic and Phonemic Cueing. *Neuropsychologia* **1991**, *29* (11), 1083–1093. https://doi.org/10.1016/0028-3932(91)90078-M.

(84) Wechsler, D. Wechsler Abbreviated Scale of Intelligence. **1999**.

(85) O'Bryant, S. E.; Lacritz, L. H.; Hall, J.; Waring, S. C.; Chan, W.; Khodr, Z. G.; Massman, P. J.; Hobson, V.; Cullum, C. M. Validation of the New Interpretive Guidelines for the Clinical Dementia Rating Scale Sum of Boxes Score in the National Alzheimer's Coordinating Center Database. *Arch Neurol* **2010**, *67* (6), 746–749. https://doi.org/10.1001/archneurol.2010.115.

(86) Tzeng, R.-C.; Yang, Y.-W.; Hsu, K.-C.; Chang, H.-T.; Chiu, P.-Y. Sum of Boxes of the Clinical Dementia Rating Scale Highly Predicts Conversion or Reversion in Predementia Stages. *Front Aging Neurosci* **2022**, *14*, 1021792. https://doi.org/10.3389/fnagi.2022.1021792.

(87) Delis, D. C.; Kramer, J. H.; Kaplan, E.; Ober, B. A. CaliforniaVerbalLearningTest–Secondedition. Adultversion. *Manual. Psychological Corporation***2000**.

(88) Woods, S. P.; Delis, D. C.; Scott, J. C.; Kramer, J. H.; Holdnack, J. A. The California Verbal Learning Test – Second Edition: Test-Retest Reliability, Practice Effects, and Reliable Change Indices for the Standard and Alternate Forms. *Archives of Clinical Neuropsychology* **2006**, *21* (5), 413–420. https://doi.org/10.1016/j.acn.2006.06.002.

(89) Delis, D. C.; Kaplan, E.; Kramer, J. H. Delis-Kaplan Executive Function System. *The Psychological Corporation* **2001**. https://doi.org/10.1037/t15082-000.

(90) Wechsler, D. *WAIS-III: Administration and Scoring Manual : Wechsler Adult Intelligence Scale*, 3rd ed.; Psychological Corporation: San Antonio, Tex, 1997.

(91) Woods, D. L.; Kishiyama, M. M.; Yund, E. W.; Herron, T. J.; Edwards, B.; Poliva, O.; Hink, R. F.; Reed, B. Improving Digit Span Assessment of Short-Term Verbal Memory. *J Clin Exp Neuropsychol* **2011**, *33* (1), 101–111. https://doi.org/10.1080/13803395.2010.493149.

(92) Dubois, B.; Slachevsky, A.; Litvan, I.; Pillon, B. The FAB: A Frontal Assessment Battery at Bedside. *Neurology* **2000**, *55*, 1621–1626. https://doi.org/10.1212/WNL.55.11.1621.

(93) Nasreddine, Z. S.; Phillips, N. A.; Bédirian, V.; Charbonneau, S.; Whitehead, V.; Collin, I.; Cummings, J. L.; Chertkow, H. The Montreal Cognitive Assessment, MoCA: A Brief Screening Tool for Mild Cognitive Impairment. *Journal of the American Geriatrics Society* **2005**, *53* (4), 695–699.

(94) Song, C.-S.; Lee, H.-S.; Chun, B.-Y. Comparison of Montreal Cognitive Assessment in Korean Version for Predicting Mild Cognitive Assessment in 65-Year and Over Individuals. *Occupational Therapy International* **2022**, *2022*, e4108434. https://doi.org/10.1155/2022/4108434.

(95) Kang, I.-W.; Beom, I.-G.; Cho, J.-Y.; Son, H.-R. Accuracy of Korean-Mini-Mental Status Examination Based on Seoul Neuro-Psychological Screening Battery II Results. *Korean J Fam Med* **2016**, *37* (3), 177–181. https://doi.org/10.4082/kjfm.2016.37.3.177.

(96) Chung, S.; Wang, X.; Fieremans, E.; Rath, J. F.; Amorapanth, P.; Foo, F.-Y. A.; Morton, C. J.; Novikov, D. S.; Flanagan, S. R.; Lui, Y. W. Altered Relationship between Working Memory and Brain Microstructure after Mild Traumatic Brain Injury. *AJNR Am J Neuroradiol* **2019**, *40* (9), 1438–1444. https://doi.org/10.3174/ajnr.A6146.

(97) Guo, Q.; Zhou, B.; Zhao, Q.; Wang, B.; Hong, Z. Memory and Executive Screening (MES): A Brief Cognitive Test for Detecting Mild Cognitive Impairment. *BMC Neurology* **2012**, *12* (1), 119. https://doi.org/10.1186/1471-2377-12-119.

(98) Arevalo-Rodriguez, I.; Smailagic, N.; Figuls, M. R. i; Ciapponi, A.; Sanchez-Perez, E.; Giannakou, A.; Pedraza, O. L.; Cosp, X. B.; Cullum, S. Mini-Mental State Examination (MMSE) for the Detection of Alzheimer's Disease and Other Dementias in People with Mild Cognitive Impairment (MCI). *Cochrane Database of Systematic Reviews* **2015**, No. 3. https://doi.org/10.1002/14651858.CD010783.pub2.

(99) Folstein, M. F.; Folstein, S. E.; McHugh, P. R. "Mini-Mental State": A Practical Method for Grading the Cognitive State of Patients for the Clinician. *Journal of Psychiatric Research* **1975**, *12* (3), 189–198. https://doi.org/10.1016/0022-3956(75)90026-6.

(100) Schoenberg, M. R.; Dawson, K. A.; Duff, K.; Patton, D.; Scott, J. G.; Adams, R. L. Test Performance and Classification Statistics for the Rey Auditory Verbal Learning Test in Selected Clinical Samples. *Archives of Clinical Neuropsychology* **2006**, *21* (7), 693–703. https://doi.org/10.1016/j.acn.2006.06.010.

(101) Osterrieth, P. A. Le Test de Copie d'une Figure Complexe; Contribution à l'étude de La Perception et de La Mémoire. [Test of Copying a Complex Figure; Contribution to the Study of Perception and Memory.]. *Archives de Psychologie* **1944**, *30*, 206–356.

(102) Shin, M.-S.; Park, S.-Y.; Park, S.-R.; Seol, S.-H.; Kwon, J. S. Clinical and Empirical Applications of the Rey–Osterrieth Complex Figure Test. *Nat Protoc* **2006**, *1* (2), 892–899. https://doi.org/10.1038/nprot.2006.115.

(103) Basso, A.; Capitani, E.; Laiacona, M. Raven's Coloured Progressive Matrices: Normative Values on 305 Adult Normal Controls. *Funct Neurol* **1987**, *2* (2), 189–194.

(104) The Raven's Progressive Matrices: Change and Stability over Culture and Time. *Cognitive Psychology* **2000**, *41* (1), 1–48. https://doi.org/10.1006/cogp.1999.0735.

(105) Marinus, J.; Visser, M.; Verwey, N. A.; Verhey, F. R. J.; Middelkoop, H. A. M.; Stiggelbout, A. M.; van Hilten, J. J. Assessment of Cognition in Parkinson's Disease. *Neurology* **2003**, *61* (9), 1222–1228. https://doi.org/10.1212/01.WNL.0000091864.39702.1C.

(106) Fujibayashi, M.; Nagatsuka, N.; Yoshida, T.; Howard, D.; Franklin, S.; Whitworth, A. Sophia Analysis of Language in Aphasia. Chiba: Test Center Co Ltd**:** Chiba, Japan, **2004**.

(107) Jensen, A. R.; Rohwer, W. D. The Stroop Color-Word Test: A Review. *Acta Psychologica* **1966**, *25*, 36–93. https://doi.org/10.1016/0001-6918(66)90004-7.

(108) Scarpina, F.; Tagini, S. The Stroop Color and Word Test. *Frontiers in psychology* **2017**, *8*, 557.

(109) Fellows, R. P.; Schmitter-Edgecombe, M. Symbol Digit Modalities Test: Regression-Based Normative Data and Clinical Utility. *Archives of Clinical Neuropsychology* **2020**, *35* (1), 105–115. https://doi.org/10.1093/arclin/acz020.

(110) Joy, S.; Kaplan, E.; Fein, D. Digit Symbol – Incidental Learning in the WAIS-III: Construct Validity and Clinical Significance. *The Clinical Neuropsychologist* **2003**, *17* (2), 182–194. https://doi.org/10.1076/clin.17.2.182.16495.

(111) Vertesi, A.; Lever, J. A.; Molloy, D. W.; Sanderson, B.; Tuttle, I.; Pokoradi, L.; Principi, E. Standardized Mini-Mental State Examination. Use and Interpretation. *Canadian Family Physician* **2001**, *47* (10), 2018–2023.

(112) Ahn, H.-J.; Chin, J.; Park, A.; Lee, B. H.; Suh, M. K.; Seo, S. W.; Na, D. L. Seoul Neuropsychological Screening Battery-Dementia Version (SNSB-D): A Useful Tool for Assessing and Monitoring Cognitive Impairments in Dementia Patients. *Journal of Korean Medical Science* **2010**, *25* (7), 1071–1076. https://doi.org/10.3346/jkms.2010.25.7.1071.

(113) Ashendorf, L.; Jefferson, A. L.; O'Connor, M. K.; Chaisson, C.; Green, R. C.; Stern, R. A. Trail Making Test Errors in Normal Aging, Mild Cognitive Impairment, and Dementia. *Archives of Clinical Neuropsychology* **2008**, *23* (2), 129–137. https://doi.org/10.1016/j.acn.2007.11.005.

(114) Salthouse, T. A. What Cognitive Abilities Are Involved in Trail-Making Performance? *Intelligence* **2011**, *39* (4), 222–232. https://doi.org/10.1016/j.intell.2011.03.001.

(115) Goetz, C. G.; Tilley, B. C.; Shaftman, S. R.; Stebbins, G. T.; Fahn, S.; Martinez-Martin, P.; Poewe, W.; Sampaio, C.; Stern, M. B.; Dodel, R.; Dubois, B.; Holloway, R.; Jankovic, J.; Kulisevsky, J.; Lang, A. E.; Lees, A.; Leurgans, S.; LeWitt, P. A.; Nyenhuis, D.; Olanow, C. W.; Rascol, O.; Schrag, A.; Teresi, J. A.; van Hilten, J. J.; LaPelle, N. Movement Disorder Society-Sponsored Revision of the Unified Parkinson's Disease Rating Scale (MDS-UPDRS): Scale Presentation and Clinimetric Testing Results. *Movement Disorders* **2008**, *23* (15), 2129–2170. https://doi.org/10.1002/mds.22340.

(116) Huang, K.-L.; Hsiao, I.-T.; Kuo, H.-C.; Hsieh, C.-J.; Hsieh, Y.-C.; Wu, Y.-M.; Wey, S.-P.; Yen, T.-C.; Lin, K.-J.; Huang, C.-C. Correlation between Visual Association Memory Test and Structural Changes in Patients with Alzheimer's Disease and Amnestic Mild Cognitive Impairment. *Journal of the Formosan Medical Association* **2019**, *118* (9), 1325–1332. https://doi.org/10.1016/j.jfma.2018.12.001.

(117) Lindeboom, J.; Schmand, B.; Tulner, L.; Walstra, G.; Jonker, C. Visual Association Test to Detect Early Dementia of the Alzheimer Type. *J Neurol Neurosurg Psychiatry* **2002**, *73* (2), 126–133. https://doi.org/10.1136/jnnp.73.2.126.

(118) Amador, J. A. La Escala de Memoria de Wechsler Cuarta Edición (WMS-IV). **2015**.

(119) Wechsler, D. A Standardized Memory Scale for Clinical Use. *The Journal of Psychology* **1945**, *19* (1), 87–95.

(120) Öktem-Tanör, Ö. Handboook of Öktem Verbal Memory Process Test (Öktem-VMPT). *Ankara: Turkish Psychological Association* **2011**.

(121) Warrington, E. K. *Visual Object and Space Perception Battery*. https://www.pearsonclinical.co.uk/store/ukassessments/en/Store/Professional-Assessments/Cognition-%26-Neuro/Visual-Object-and-Space-Perception-Battery/p/P100009236.html (accessed 2024-01-14).
(122) Lezak, M. D. *Neuropsychological Assessment*; Oxford University Press, 2004.
(123) Shao, Z.; Janse, E.; Visser, K.; Meyer, A. S. What Do Verbal Fluency Tasks Measure? Predictors of Verbal Fluency Performance in Older Adults. *Front Psychol* **2014**, *5*, 772. https://doi.org/10.3389/fpsyg.2014.00772.
(124) Teng, E. L.; Chui, H. C. The Modified Mini-Mental State (3MS) Examination. *J Clin Psychiatry* **1987**, *48* (8), 314–318.
